# Mechanism of Action of Cyclophilin A Explored by Metadynamics Simulations

**DOI:** 10.1371/journal.pcbi.1000309

**Published:** 2009-03-13

**Authors:** Vanessa Leone, Gianluca Lattanzi, Carla Molteni, Paolo Carloni

**Affiliations:** 1 International School for Advanced Studies (SISSA), Trieste, Italy; 2 IIT—Italian Institute of Technology and DEMOCRITOS, Trieste, Italy; 3 University of Bari, Bari, Italy; 4 TIRES and INFN, Bari, Italy; 5 Department of Physics, King's College, London, United Kingdom; University of Houston, United States of America

## Abstract

*Trans/cis* prolyl isomerisation is involved in several biological processes, including the development of numerous diseases. In the HIV-1 capsid protein (CA), such a process takes place in the uncoating and recruitment of the virion and is catalyzed by cyclophilin A (CypA). Here, we use metadynamics simulations to investigate the isomerization of CA's model substrate HAGPIA in water and in its target protein CypA. Our results allow us to propose a novel mechanistic hypothesis, which is finally consistent with all of the available molecular biology data.

## Author Summary

Peptidyl prolyl isomerases are ubiquitous enzymes whose actions are crucial in several biological processes, such as, for instance, in cellular signalling and in the onset of several diseases, e.g., HIV infection. Therefore, these isomerases are promising targets for the design of new drugs. For this purpose, we need to understand their molecular mechanism of action. One of the most characterized peptidyl prolyl isomerases is cyclophilin A. Previous studies characterized the roles of several protein regions in isomerase function. However, there are still experimentally identified important portions of the protein whose specific actions in the mechanism are still not known. Here, we address this problem by an extensive computational study of cyclophilin A and a substrate peptide that is part of the HIV-1 capside protein. We present a novel four-step mechanism of the whole enzymatic process, which is consistent with all of the available experimental data. Moreover, these steps can be used as targets for the development of drugs, e.g., for HIV-1 infection.

## Introduction

Proline *trans/cis* isomerization takes part in several fundamental biological processes, including protein folding [1]–[3], immune response [3],[4], ion channel gating [5] and cellular signalling [3],[4],[6]. The process, which is also associated to the development of a variety of diseases including HIV-1 infection [7],[8], carcinogenesis [6] and Alzheimer's [9], is catalyzed by prolyl isomerase enzymes [1],[10],[11].

**Figure 1:**
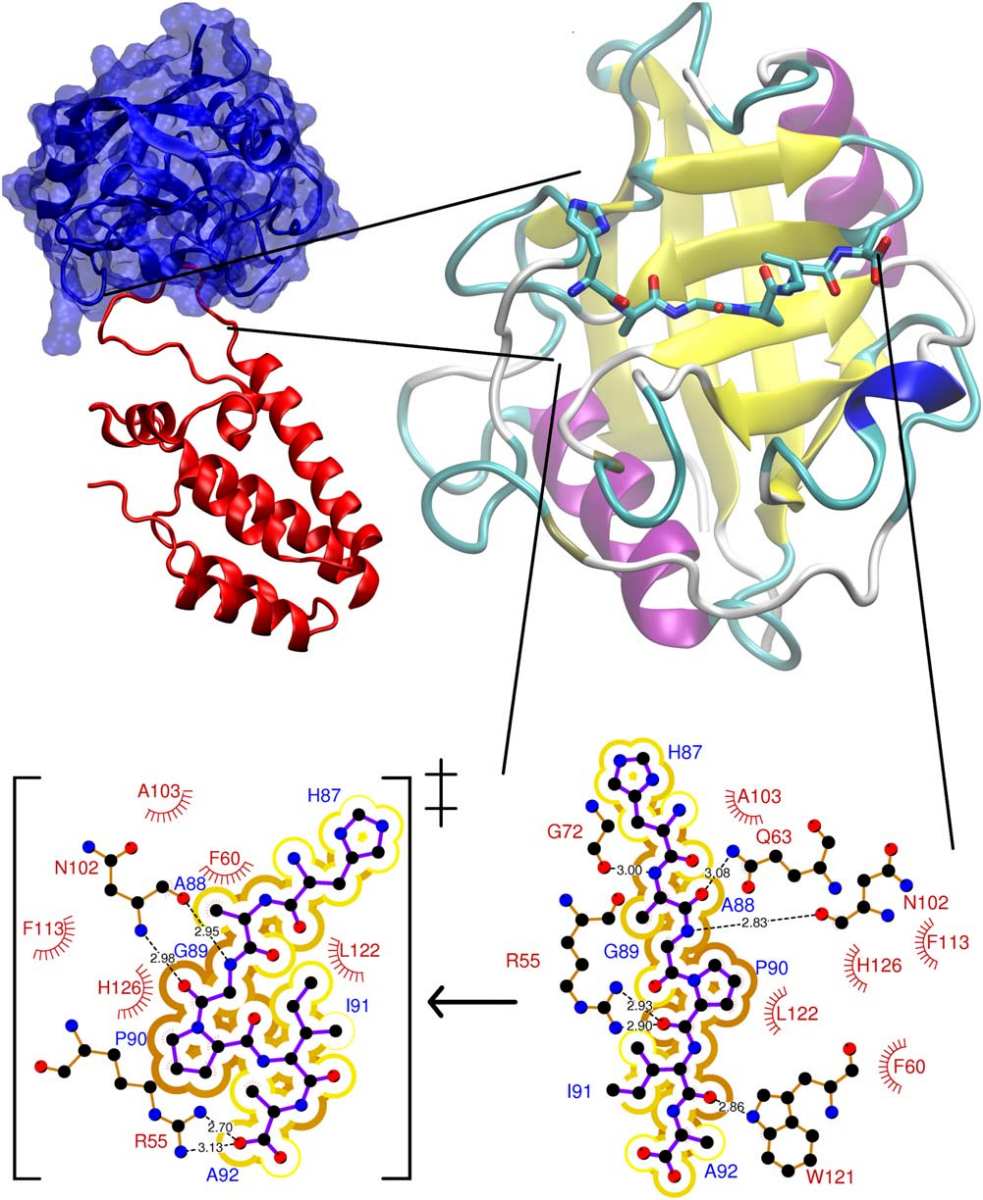
Human CypA in complex with CA. Human CypA is a cytoplasmatic single peptide chain, 165 amino acids long. *Top:* The X-ray structure shows that it is a β-barrel with eight antiparallel β-strands and two α-helices flanking the β-barrel [13]. These secondary structures are connected by several flexible loops. The β-barrel contains the active site for cis/trans prolyl isomerization. The CA protein is composed by two helices connected by a loop. This contains the G89-P90 target peptide bond (*Bottom*, *right*). *Bottom*, *left:* Transition state of the *cis-trans* isomerisation, emerging from this and previous [14],[18],[22] calculations.

The best characterized isomerization *in vivo* and *in vitro* occurs in the uncoating and recruitment of the human HIV-1 capsid (CA) protein in the virions [7], and it is catalyzed by cyclophilin A isomerase (CypA, [12]). At the structural level, CypA features α-helices flanking a beta-barrel, while CA is made of several α-helices connected by loops (Figure 1). In the X-ray structure of the complex [13], the targeted proline-containing backbone unit (G89-P90), is accommodated in a hydrophobic pocket of CypA (residues F60, F113, L122, and H126 in Figure 1). Although the backbone unit is in *trans* conformation in the X-ray structure [13], NMR studies have shown that 45% of conformers are *cis* for CA-CypA^R55A^ in aqueous solution [12]. The rather significant population of *cis* conformers could arise not only by the replacement of R55 with Ala, but also by crystal packing forces, different temperature conditions (100 K and 298 K for the X-ray and NMR experiments, respectively), along with different hydration and ionic strength in the two experiments. Free energy calculations further support the hypothesis that CypA promotes a significant population of *cis* conformation [14],[15]. Thus, the *cis* population is likely to increase substantially from water – where it is ∼10% [12] – to the complex in aqueous solution.

*In vitro* kinetic measurements (Table 1) show that CypA decreases the isomerization free energy barrier (ΔG^‡^_cis→trans_, ΔF^‡^_cis→trans_) of modified substrate fragments in solution by ∼7 kcal/mol [16],[17]. Free energy calculations, based either on classical [15] or quantum-mechanical/molecular mechanics (QM/MM) [14], of the 6 aminoacids long substrate fragment point to a similar trend (Table 1). In addition, they suggest that H-bonding between the backbone unit of the targeted glycine-proline peptidyl bond and that of N102@CypA [14],[15],[18], as well as van der Waals interactions between substrate and the CypA hydrophobic pocket (F60, F113, L122, and H126) stabilize the transition state (TS) [14],[15],[18]. These hypotheses are consistent with the decrease of k_cat_/K_m_ associated with the polymorphism of cyclophilins in 102 position (N to T, S, H and R, Table S1) and in the F60A, F113A and H126Q CypA mutants [19],[20] (Table S1). NMR studies, along with computations, have further suggested that the motions of several CypA residues (including R55 and N102) are linked to the enzymatic activity [15], [21]–[23]. Free energy calculations characterized a network of protein vibrations in CypA that are associated with its isomerase activity: flexible loops on the surface are connected to the active site by a network of hydrogen bonds [15],[22],[23].

**Table 1:** Available *in vitro* experimental and theoretical thermodynamic data (kcal/mol) associated to the *cis/trans* isomerisation of peptides binding to CypA protein with the values reported for the peptide in water and bound to the protein (bold face)^a^.

Source of Data	Isomerized Peptide	Thermodynamic Parameter	Values (kcal/mol)	Ref
**Experiment**	SUC-Ala-Phe-Pro-Phe-pNA^b^	**ΔG°_cis→trans_** c	0.6 **−0.2**	[17]
	SUC-Ala-Phe-Pro-Phe-pNA^b^	**ΔG^‡^_cis→trans_** d	20.1 **12.9**	[17]
	SUC-Ala-Ala-Pro-Phe-pNA^b^	**ΔG^‡^_cis→trans_** d	19.3 **10.8**	[16]
**Theory**	/His-Ala-Gly-Pro-Ile-Ala/f	**ΔF_cis→trans_** e	1.2 **−0.1**	[14]
	/His-Ala-Gly-Pro-Ile-Ala/f	**ΔF^‡^_cis→trans_** d **(clockwise)** h	18.5 **11.8**	[14]
	/His-Ala-Gly-Pro-Ile-Ala/f	**ΔF^‡^_cis→trans_** d **(counterclockwise)** h	18.9	[14]
	/His-Ala-Gly-Pro-Ile-Ala/g	**ΔF_cis→trans_** e	1 **2**	[15]
	/His-Ala-Gly-Pro-Ile-Ala/g	**ΔF^‡^_cis→trans_** d **(clockwise)** h	12 **9**	[15]
	/His-Ala-Gly-Pro-Ile-Ala/g	**ΔF^‡^_cis→trans_** d **(counterclockwise)** h	12 **7**	[15]

a For *in vitro* experiments ΔF is about equal to ΔG [10].

b Thermodynamic properties for a peptide having the CypA target sequence have not been determined experimentally neither in CypA nor in water solution.

c Free energy differences between *trans* and *cis* minima at standard condition.

d Activation free energies. The experimental ΔG**^‡^** values are converted from kinetic data for SUC-Ala-Phe-Pro-Phe-pNa in CypA using Transition State Theory with a prefactor of k_B_T/h (T = 283 K for [17], T = 273 K for [16]).

e Free energy differences between *trans* and *cis* minima.

f SCC-DFTB/TIP3P umbrella sampling calculation. The free energy is calculated as a function of τ (C^i-1^-O^i-1^-Cδ^i^-Cα^i^) similar to ζ (Cα^i-1^-O^i-1^-Cδ^i^-Cα^i^), see Figure 2.

g Amber force field umbrella sampling calculation using ω in NVE ensemble (Figure 2).

h Cα^i-1^ respective to C(i) is in the same side (clockwise) or in the opposite site (counterclockwise) of proline ring (Figure 2).

In spite of the great progress in describing the catalytic process, key mechanistic issues need to be addressed. Kinetic studies [17] suggested that the overall process involves *trans* and *cis* forms in solution and in complex with the protein. However, the studies so far consider mostly TS stabilization. Most importantly, the current proposed mechanism cannot explain a plethora of molecular biology data. These studies show that residues *not* involved in TS stabilization in the proposed mechanisms are important for the function, as their mutations cause a decrease of k_cat_/K_m_. Indeed, k_cat_/K_m_ passes from 1.6 10^7^ M^−1^ s^−1^ in the wild type to 1.6 10^4^ M^−1^ s^−1^ by mutating the fully conserved R55 residue with Ala [20]. Although this mutation was proposed originally to destroy an H-bond stabilizing uniquely the TS [13],[24], such H-bond was subsequently ruled out in computational works [14],[15],[18] and so far no functional role has been ascribed to R55. In addition, the H54Q mutation, along with the I57V polymorphism, causes a decrease of k_cat_/K_m_, although these residues are not involved in TS stabilization (Table S1).

Here we use molecular simulations to address these issues. We calculate the free energy associated with the isomerisation of the 6 aminoacids long (/HAGPIA/) substrate fragment in water and in complex with CypA. The free energy is calculated as a function of the two reaction coordinates ζ and ψ (defined in Figure 2), which have been suggested to describe best the energetics of the process [25], as well as other pairs of different coordinates to cross-check our results. We use here metadynamics [26] (in its bias exchange extension [27]), which has already been employed to predict the energetics of protein/peptide interactions [28]. The potential used is an effective one (specifically the AMBER99 force field [29]). This choice allows a very efficient sampling because of its relatively small computational cost. In spite of its limitations, [30] this force field is expected to be relatively accurate to describe equilibrium conformations, which is a key aspect of our problem. The accuracy of the force field for minima and transition states is assessed by comparing our results with first principle quantum chemistry calculations. Based on this comparison, we find that the force-field biases on the energetics of the minima are negligible, whereas their influence on the barriers is more significant. Therefore, here the free energy differences between minima are used to predict the relative populations of the equilibrium conformations, whilst the calculated barriers are only compared at the qualitative level to discriminate the most likely *cis/trans* isomerisation path.

**Figure 2:**
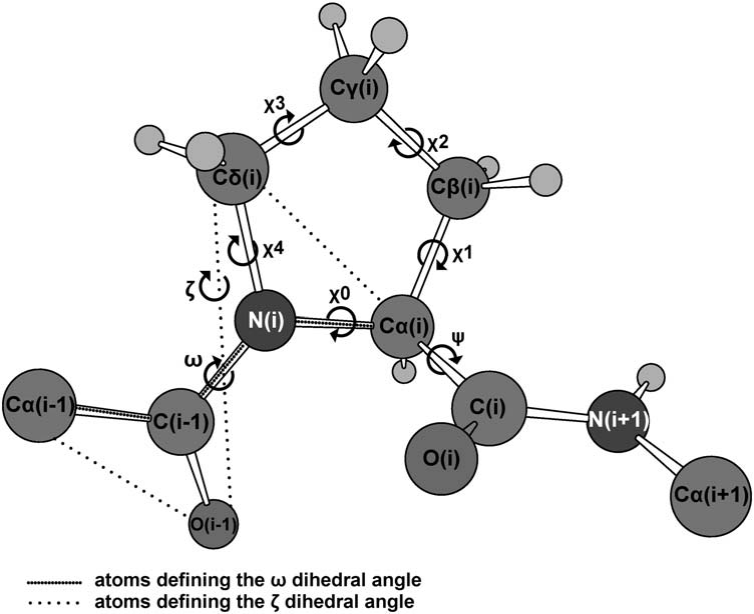
Dihedral angles in prolyl isomerisation. The dihedral angles ω and ζ identify the *trans* (∼±180°) or *cis* (∼0°) conformation. The angle ψ determines the rotation along Cα(i)-C(i) bond. The dihedral angles χ^0^, χ^1^, χ^2^, χ^3^ and χ^4^ measures the puckering of the proline ring.

The enzyme turns out to dramatically stabilize the populations of one specific *cis* conformer relative to the *trans* ones, which instead are by far the most stable in aqueous solution. In addition, it lowers the free energy barrier of a specific, one–way isomerization from *trans* to *cis*. These findings allow us to propose a mechanistic hypothesis for the isomerization process that is consistent with *all* the available experimental data.

## Methods

### Structural Models and MD Calculations

The initial structural models of /HAGPIA/ in the free state (PEPT-WAT) and in complex with CypA (PEPT-CypA) were obtained from the CA N-terminal domain/CypA X-ray structure at 2.0 Å resolution (PDBID:1M9C) [13]. The X-ray data were collected at 100 K and slightly basic pH (8.0) [13]. The peptide and the protein were considered in their zwitterionic form. The protonation states of all histidine residues were determined by pKa calculations based on the H++ server [31],[32] and by visual inspection of the hydrogen bond network. All His residues were protonated at Nδ1. The result for PEPT-WAT is consistent with the NMR study on CA N-terminal domain at pH 7 [33].

PEPT-WAT and PEPT-CypA were solvated in a 32 Å×37 Å×38 Å and 57 Å×80 Å 60 Å boxes, containing 1,423 water molecules (for a total of 4,349 atoms) and 8,233 water molecules and a Cl^−^ ion (for a total of 27,283 atoms), respectively. The ion was added to neutralize the system.

The Amber99 all atom force field [29] was used for the protein and the chlorine atom; the TIP3P model [34] was used for water. After minimization up to a convergence of 10^−4^ kcal/mol (conjugate gradient algorithm), the system was equilibrated for 1 ns (time step of 1 fs) in an isothermal-isobaric ensemble (1 atm, 310 K) with the Langevin barostat [35] (the oscillation period and the decay coefficient were set to 200 fs and 100 fs, respectively) and thermostat [36] (coupling coefficient 1 ps^−1^). We use the Particle Mesh Ewald scheme [37] with 12 Å cutoff and 0.75 Å-spaced Fourier grid; we assume a dielectric constant of 1. Van der Walls interaction cutoff was set to 12 Å. Minimization and equilibration were performed with the NAMD 2.6 program suite [38].

### Free Energy Calculations of Proline *cis/trans* Isomerisation

Free energies were calculated using the metadynamics method in its bias exchange variant [26],[27]. This approach consists in a continuous addition of a history dependent potential energy that enforces the dynamics to explore conformations that were not previously visited. Briefly, the forces acting on each atom, or centroid, are corrected by a history dependent contribution, obtained as the derivative of the history dependent potential energy *V*_t_ with respect to the atom coordinate. *V_t_* is given by the sum of a set of Gaussian functions centered on the values *s_t_* of each chosen collective variable (CV) *s* as time *t*:
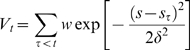
(1)The time interval between the addition of two Gaussian functions *τ*, as well as the Gaussian height *w* and Gaussian width *δ*, are tuned to optimize the ratio between accuracy and computational cost (Table S2). Eventually, after exploring all conformations defined within the CV space, the probability distribution of Gaussians becomes flat, and the free energy profile does not change any more. At this stage, the free energy surface can be easily reconstructed as the opposite of the sum of all Gaussians.

Here we use the bias exchange variant of this method [27], in which several trajectories 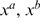 with different history dependent potentials (for instance two CVs *a* and *b*), 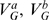, are run in parallel. At specific time intervals (of the order of few ps), 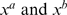 are swapped with a probability 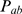 evaluated by the standard Metropolis scheme [39].
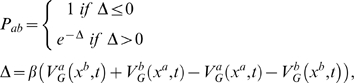
(2)where k_B_ is the Boltzmann constant and T is the temperature. This method was shown to provide an efficient sampling of the conformational space [27].

The free energies associated with *cis/trans* isomerization of the G3P4 bond in PEPT-WAT and PEPT-CypA were calculated as a function of pairs of CVs in the canonical ensemble. The free energies were first calculated as a function of the ζ and ψ dihedral angles (Figure 2 and Table S2), because these angles have been shown to be essential to describe the isomerisation process [25]. The resulting free energy plot was used to define the minima and the transition state regions (*R* hereafter). Briefly, we performed a cluster analysis of all the structures [40] and evaluated their population using an umbrella sampling-like approach [41]; minima and TSs are characterized by several clusters and only one cluster, respectively (See Text S1 for details). Free energy barriers were then calculated as differences between the TS cluster and the lowest cluster in the basin of the minimum.

The sampling of TSs was higher in PEPT-WAT than in PEPT-CypA, as shown by the few number of structures clusterized in TSs' *R* (i.e. less than 10 structures). To improve sampling in PEPT-CypA, we performed additional 4 ns-long MD simulations in which the ζ and ψ angles were harmonically restrained at the values of the TSs. Restraint center and their associated force constants were fitted so as to keep the entire MD within the region of interest.

**Figure 3:**
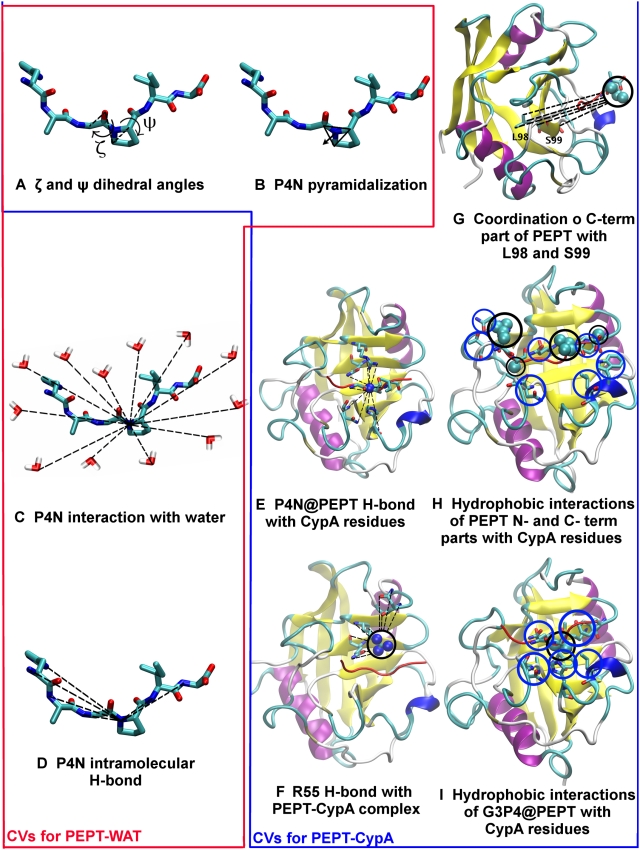
Collective variables. Collective variables used in bias exchange metadynamics simulations. The red box encloses the variables used in the PEPT-WAT system, the blue box contains variables used in PEPT-CypA. We define briefly each CV as follows: (A) see Figure 2; (B) Proline nitrogen pyramidalization is defined as the distance between P4N atom and the center of the plane determined by G3C-P4Cα-P4Cδ; (C) P4N interaction with water is defined as P4N coordination with O atoms of all water molecules; (D) P4N intramolecular H-bond was defined as P4N coordination with N(-H) atoms of all H-bonds donor residues of PEPT; (E) P4N@PEPT H-bond with PEPT or CypA was described as the coordination of P4N with N(-H) and O(-H) atoms of all possible H-bond donors of PEPT and CypA; (F) R55 H-bond with PEPT-CypA is illustrated as the coordination between R55@CypA and CypA active site residues; (G) Interaction of L98 and S99 with C-terminal part of the substrate is defined as the coordination between non polar carbons atoms of C-terminal@PEPT and those of L99 S99; (H) Hydrophobic interactions between the N and C terminal of PEPT with the active site were described as the coordination of the non polar carbons of the PEPT N-and C-terminal with non polar carbons of the residues in the active site of CypA; I) Non-polar carbons coordination of G3P4@PEPT with CypA active site residues.

To test the robustness of our insights on the enzyme mechanism obtained by the above free energy profiles, we compared our results with free energy plots as a function of other pairs of CVs. These are: (i) the ζ angle and the proline nitrogen (P4N) pyramidalization *p* that might have a role in peptidyl prolyl *cis/trans* rotation [25]. *p* is defined as the distance between P4N and the centre of a plane determined by three atoms belonging to G3 and P4 (Figure 3 and Table 2). (ii) The ζ and P4N H-bond coordination number (*s1*) that has been shown to be important for *in vacuo* prolyl isomerization [25]:
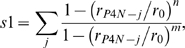
(3)where *r*_P4N-j_ is the distance between P4N and the atom *j* (excluding solvent molecules) while *r_0_*, *n*, *m* are parameters chosen to obtain *s1*∼1, when the P4N forms one H-bond (Table S2). *s1* increases with the number of H-bonds formed by P4N with H-bond acceptors of the peptide (in water) or of the peptide and protein in the complex.

(iii) *p* and *s1*, to assess the correlation between the pyramidalization and H-bonding of the P4N atom.

**Table 2:** Minima and TSs population in all the CV pairs containing the dihedral angle ζ.

System	CV pairs	*trans_0_*	*cis_0_*	*trans_180_*	*cis_180_*	TS1	TS2	TS3	TS4
**PEPT-WAT**	**Best fit**	**94.0%**	5.0%	0.6%	6.3 10^−5^%	6.7 10^−8^%	3.9 10^−10^%	8.0 10^−9^%	5.7 10^−11^%
	**1**	95.6%	4.1%	0.3%	2.0 10^−5^%	5.2 10^−8^%	4.3 10^−10^%	7.0 10^−9^%	2.3 10^−11^%
	**2**	91.8%	5.2%	2.7%	0.2%	5.4 10^−8^%	2.4 10^−10^%	1.0 10^−8^%	4.5 10^−11^%
	**3**	94.1%	4.0%	1.8%	1.0 10^−1^%	9.0 10^−8^%	2.1 10^−10^%	6.3 10^−9^%	1.5 10^−10^%
	**4**	90.6%	5.6%	3.6%	0.1%	7.1 10^−8^%	1.2 10^−9^%	5.4 10^−9^%	-
**PEPT-CypA**	**Best fit**	5.2%	0.2%	18%	**76.2%**	7.1 10^−10^%	4.3 10^−8^%	1.7 10^−12^%	1.5 10^−10^%
	**1**	2.0%	0.6%	8.4%	89.1%	1.5 10^−9^%	3.0 10^−8^%	3.1 10^−11^%	7.8 10^−11^%
	**2**	10.2%	0.1%	25.0%	64.6%	2.1 10^−9^%	1.7 10^−8^%	-	9.3 10^−11^%
	**3**	15.0%	1.1%	17.0%	66.9%	2.2 10^−9^%	2.6 10^−8^%	-	9.7 10^−11^%
	**4**	4.3%	3.7%	40.2%	52.7%	4.9 10^−10^%	4.1 10^−8^%	2.4 10^−13^%	2.1 10^−10^%
	**5**	0.8%	0.3%	16.0%	76.4%	4.6 10^−10^%	4.2 10^−8^%	6.4 10^−12^%	1.6 10^−10^%
	**6**	0.7%	0.5%	16.0%	76.3%	2.8 10^−9^%	6.3 10^−8^%	-	1.7 10^−10^%
	**7**	3%	3.2 10^−2^%	19.6%	76.8%	2.3 10^−10^%	2.6 10^−8^%	-	-

Finally, we calculated the free energy profiles as a function of selected pairs specific for PEPT-WAT or for PEPT-CypA. For PEPT-WAT, these are the following: (*i*) the ζ and P4N coordination number with water solvent molecules (*s2*). *s2* is defined as *s1* except that *j* runs over the water oxygen atoms. This pair tests whether water is able to catalyze prolyl isomerization by forming an H-bond with P4N. (*ii*) *p* and *s2*, to evaluate if there is a correlation between P4N pyramidalization and its H-bonding to water molecules.

For PEPT-CypA, we introduced CVs that relate the substrate to the enzyme: *i*) hydrophobic coordination numbers *s3*, *s4* and *s5*, i.e. quantities which depend only on non-polar carbon atoms. *s3*, *s4* and *s5* describe the hydrophobic interaction between the peptide and the protein:
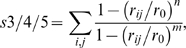
(4)where *r_ij_* is the distance between atoms *i* and *j*. *i* runs over the residues involved in the rotating prolyl bond of the peptide (G3P4) for *s3*, and the C and N terminal of the peptide (H1, A2, I5 and A6) for *s4*; *j* runs over any non-polar carbon of CypA belonging to residues located at 4 Å or less from the peptide (Table S2) for both *s3* and *s4*. The parameters *n* and *m* were chosen so as to distinguish conformations with and without hydrophobic interactions (Table S2). This was done by running 1 ns MD test simulations with different *n* and *m* parameters.

*s5* describes a subset of such hydrophobic interactions, which play a particularly important role during the isomerisation, as suggested by NMR studies [21]. These are the interactions of L98 and S99 in CypA and C terminal of the peptide (I5, A6). Thus, for *s5*, *i* runs over I5 and A6, while *j* runs over the atoms of L98 and S99.

Finally, we introduced the reaction coordinate *s6*:
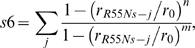
(5)where *j* runs over all the H-bond donors/acceptors of the peptide or of CypA that were found within 3Å from R55 N atoms in 1 ns MD: those turned out to belong to residues Q63 and N149; *r_R55Ns-j_* is the distance from any N atom of R55 and any atom *j*; *r_0_*, *n* and *m* are chosen as in *s1* (Table S2). This reaction coordinate increases with the number of H-bonds formed with R55 and hence measures the ability of this residue to form H-bonds with the peptide or CypA residues (organizing its active site): this has been indicated as crucial for the catalysis [14].

The free energy is calculated as a function of *(i)* ζ, *s3*; *(ii)* ζ, *s4*; *(iii)* ζ, *s5* and *(iv)* ζ, *s6* to analyze hydrophobic (*s3*, *s4*, s5) and hydrophilic (s6) interactions along with the *cis* (ζ∼0°)↔*trans*(ζ∼±180°) interconversion.

All metadynamics calculations (17 ns for PEPT-WAT and 40 ns for PEPT-CypA) were performed at the physiological temperature of 310 K. The temperature was controlled by a Nosè-Hoover thermostat [42], with coupling time constant of 0.05 ps. Electrostatic interactions were assessed using the particle mesh Ewald schemes [37] with 34 wave vectors in each dimension and fourth-order cubic interpolation. Van der Waals interactions were evaluated as specified for the equilibration phase. The time-step was 1 fs. The Gaussians were added with a frequency of 2 GHz and they had a height of 2.5 kJ/mol, the width (*δ*) for each CV is reported in Table S2. The exchange trial frequency was 400 MHz. All bonds were constrained with the LINCS algorithm [43]. We removed the protein centre of mass translation every 10 steps of molecular dynamics. The calculations were performed with a locally modified version of Gromacs 3.3.1 [27],[44],[45].

### Calculated Properties at Minima and TSs

*The number of hydrogen bonds* (*N_HB_*) was calculated in TS and minima *R*, supposing a Boltzmann distribution within the cluster, as assumed in [46] for enthalpy calculations:
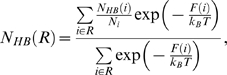
(6)where *R* is the region identifying the minima or the TS (See Figure S1), *N_i_* is the number of structures belonging to cluster *i* (see Figure S1), *N_HB_(i)* is the number of H-bonds and *F(i)* is the free energy for cluster *i*. The H-bond between the acceptor P4N and donors D is assumed to exist if the distance (D-P4N)≤3.2 Å and the angle (P4N…D-H)≤70°.

*The populations of puckered conformations* were evaluated in terms of χ^2^ (dihedral angle Cα-Cβ-Cγ-Cδ in Figure 2): up-puckering is defined when χ^2^>10°, planar puckering when -10°<χ^2^<10°, down puckering when χ^2^<−10° [47].

The population of puckered conformations P_χ^2^(C)_ is defined as:
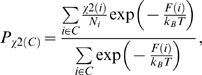
(7)where *C* = (up, down or planar puckering) and *i* is a cluster belonging to this state. The free energy associated with each conformation *C* is estimated as 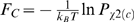.

*The interface coordination number* (*IC*) in PEPT-CypA is defined as 
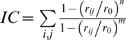
, where *i* is the index of the carbon atoms and *j* runs over the carbon atoms of CypA hydrophobic residues within 4 Å of G3 and P4 residues.

The *internal energy/entropy contributions* are evaluated for the restrained MD simulations of each minima and TS. The internal energy contribution to the free energy is calculated as the potential energy averaged over all conformations of the cluster [46], while the entropic contribution is obtained by the standard thermodynamic relation: 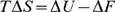.

Principal component analysis (PCA) [48],[49] was used to identify large-scale collective fluctuations in minima and TSs restrained dynamics. This analysis was performed on Cα of PEPT-CypA complex, using Gromacs 3.3.1 [44],[45]

### Errors

The statistical error of the free energy for minima and TSs are estimated as the largest difference between the F(ζ) values calculated from different 2D free energy profiles (for more details see text S1). The statistical error on the enthalpy of minima and TSs was estimated similarly. The accuracy of the force-field used (Amber [29]) was established by a comparison with quantum chemical results on a model system. We compared the *cis*↔*trans* isomerization potential energy of a N-acetyl proline methylamide using the Amber force field [29] and DFT calculations at the B3LYP/6-31G(d) [50]–[53] level of theory (see text S1 for more details on these calculations). Classical calculations were performed with Gromacs 3.3.1 [44],[45]. DFT calculations were performed with Gaussian98 [54].

## Results

We calculated the free energy F associated with the isomerization of the G3P4 bond in the /HAGPIA/ peptide as a function of the angles ζ and ψ (defined in Figure 2), which are fundamental collective variables (CVs) to describe the isomerization process [25].

The peptide is either solvated in water (PEPT-WAT) or in complex with CypA, in aqueous solution (PEPT-CypA). Force-field based metadynamics in its bias-exchange variant, in the canonical ensemble, provided the F = F(ζ, ψ) profile, from which we defined minima and TS regions and clusters within these regions (See Methods Section).

### PEPT-WAT

**Figure 4:**
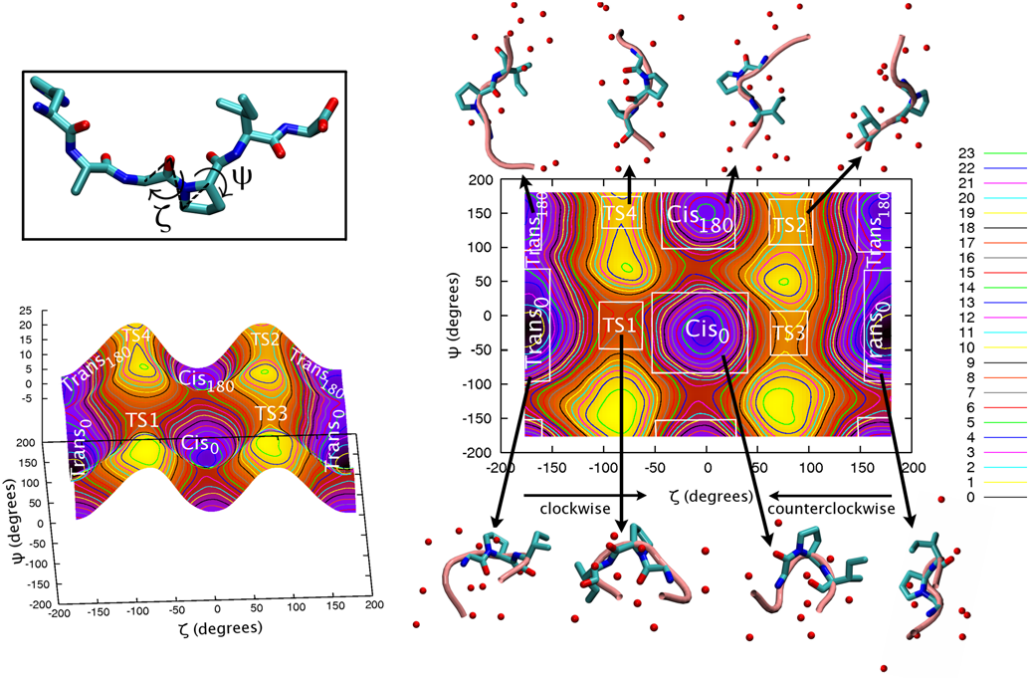
PEPT-WAT. Free energy (kcal/mol) of the /HAGPIA/ peptide in solution as a function of the dihedral angles ζ and ψ (in degrees, showed in the inset). The plot is divided in transition and minima regions, with representative structures (center of the lower free energy cluster within each minima and TS region) for each region explicitly shown (See Text S1 for details), along with water molecules within a shell of 2 Å (red spheres).

The free energy profile plot shows that the *trans_0_* conformer, in which ζ∼±180° and ψ∼0°, is the absolute minimum (Figure 4 and Table 2). The second *trans* minimum is *trans_180_* (ζ∼±180° and ψ∼±180°), whose free energy is higher by 3 kcal/mol (Table 2, Table 3), possibly because the P4N forms a H-bond to the amide group of the adjacent residue only in *trans_0_* (Table S3, see Figure 2 and Figure 1 for atom labelling).

**Table 3:** Free energy (kcal/mol) of PEPT-WAT and PEPT-CypA prolyl *cis/trans* isomerization.

System	Pathway along:	TS1	TS2	TS3	TS4
**PEPT-WAT**	*trans→cis*	14	16	18	17
	*cis→trans*	11	14	15	15
**PEPT-CypA**	*trans→cis*	14	12	18	16
	*cis→trans*	12	13	16	17

However, the number of waters around the peptide is larger for *trans_180_* than for *trans_0_*, since the former has a more extended structure (Figure 4). This water-peptide interaction may not stabilize enough *trans_180_* against *trans_0_* to overcome the stabilization of the latter by the P4N…I5N intramolecular H-bond.

The free energies of the two *cis* conformers, (*cis_0_*: ζ∼0° and ψ∼0° and cis*_180_*: ζ∼0° and ψ∼180°) are 3 and 5 kcal/mol higher than *trans_0_*, respectively. The stabilization of *trans* relative to *cis* has been ascribed to steric clashes in the *trans* conformations (see, e.g. [10]), and is affected by intramolecular interactions. In our case, in *trans* A6 H-binds to H1, A2 and G3 with higher persistence than *cis* (Table S3). This is expected to stabilize further the *trans* conformation. The reason why *cis_0_* is more stable than *cis_180_* is again the presence of the P4N…I5N H-bond only in the first conformation.

As in the case of *trans* conformers, there are more waters around the peptide in the *cis_180_* than in the *cis_0_* conformation, but water-peptide interactions do not stabilize significantly the cis*_180_* conformation compared to the intramolecular H-bond interaction that stabilize *cis_0_*.

The difference in hydration between *trans_180_* and *cis_180_* is reproduced between *trans_0_* and *cis_0_*. A direct comparison of our calculation with classical and QM/MM umbrella sampling studies based on the reaction coordinates ω [15] and τ [14], respectively, is not possible, because these calculations do not distinguish between *cis* and *trans* conformations at ψ = 0° and ψ = 180°.

The *trans_X_*→*cis_X_* and *cis_X_*→*trans_X_* (X = 0°,180°) isomerization free energy barriers range between 4–18 and 11–15 kcal/mol, respectively (Table 3). These values are similar to those obtained by force-field based umbrella sampling calculations performed on the ω variable (Table 1) [15]. However, early quantum gas phase calculations showed that, at variance with the ζ angle, the ω angle alone does not describe properly the proximity to a saddle point conformation [25].

The water shell does not change along the *trans_X_*↔TS↔*cis_X_* pathways (Figure 4). This is consistent with the suggested poor solvent reorganization during isomerisation in peptides of similar size [55],[56]. However, the solvent could play an important role for the isomerization of peptides smaller than that considered here.

The isomerisation is enthalpy-driven (Figure S2) similarly to that found experimentally on different proline containing peptides [52],[53].

Furthermore, proline puckering populations are fully consistent with statistical distributions across peptidyl prolyl bonds in the Brookhaven Protein Databank [47] (see Text S1 and Figure S3).

The lowest pathway is the *cis_0_*→TS1→*trans_0_* pathway with an associated barrier of 11 kcal/mol (Figure 4), also shown by *in vacuum* DFT potential energy calculations of N-acetylproline methylamide isomerization (see Text S1 and Table S4). This value is of the same order of the experimental values for smaller peptides (∼4 residues, Table 1). The stabilization of TS1 (as opposed to TS2-TS4) may be caused, at least in part, by the larger persistency of the P4N-I5N H-bond (Table S3 and Table S5). The formation of this H-bond, not only for TS1 but also for TS3, has been previously predicted [25]. P4N instead does not interact with the solvent (See Text S1).

Obviously, the *cis_0_*→*trans_0_* pathway is favored over the reverse one, since *trans_0_* is the most stable state.

The isomerizations involving states with ψ∼±180° are higher in free energy because the P4N-I5N intramolecular H-bond is not present in these conformation (Figure 4).

Again, a comparison with previous free energy calculations in aqueous solution is not possible since previous studies did not discriminate between *trans/cis_0_* and *trans/cis_180_*.

A similar picture emerges from calculations of the free energy as a function of other CV pairs (Table 2). These include the P4N pyramidalization, the key H-bond between P4N and the H-bond donors of the peptide (Figure 3), and P4N hydration (See Methods, Text S1 and Figure S4).

We conclude that *trans_0_* is the most populated state (Figure 4 and Table 2) and that the fastest kinetic process produces this isomer, starting from the most populated *cis* isomer (*cis_0_*).

### PEPT-CypA

**Figure 5:**
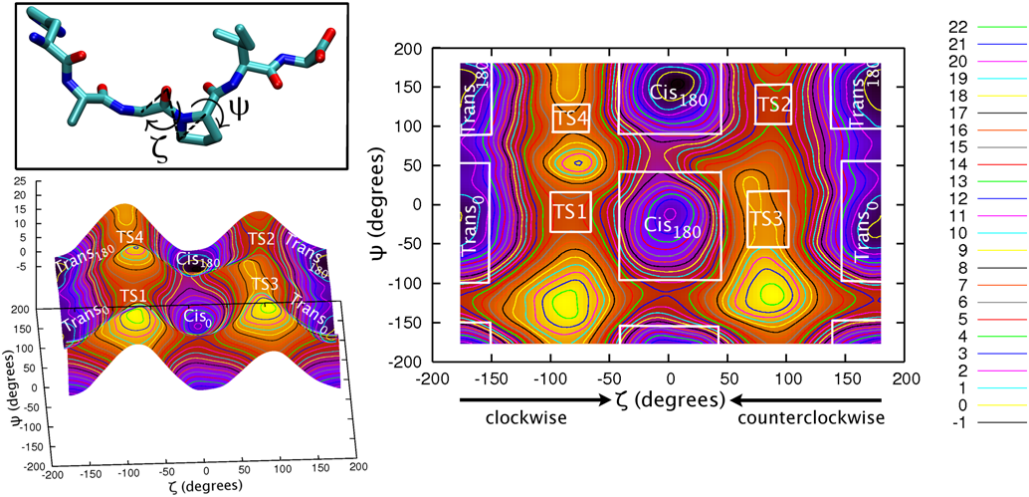
PEPT-CypA. Free energy (kcal/mol) of the /HAGPIA/ bound to CypA as a function of the dihedral angles ζ and ψ (in degrees). The plot is divided in transition and minima regions (see Text S1 for details). Representative structures of each minima and TS conformation (center of the lower free energy cluster within each minima and TS region) in PDB format are given in Datasets S1, S2, S3, S4, S5, S6, S7, S8.

The presence of the protein alters dramatically the population of the four minima in the F = F(ζ, ψ) plot (Figure 5). The global minimum, and therefore the most populated state, is not a *trans* configuration: it is *cis_180_* (representative structure in dataset S4). *Cis* stabilization has been already found by AMBER-based and QM/MM free energy calculations as a function of ω [15] and τ [14] (τ dihedral angle – defined as C_(i-1)_-O_(i-1)_-C_δ(i)_-C_α(i-1)_ – is similar to ζ – C_α (i-1)_-O_(i-1)_-C_(i) δ_-C_α (i-1)_ –used in this work). *Trans_0_* (representative structure in dataset S1) and *trans_180_* (representative structure in Dataset S2) are 1 kcal/mol higher in free energy than *cis_180_*; *cis_0_* (representative structure in Dataset S3) is scarcely populated (Figure 5 and Table 2).

*Cis_180_* features highly persistent H-bonds between N102@CypA…G3(O/N)@PEPT (Table 4) as well as hydrophobic interactions between G3P4@PEPT and N102, Q63@CypA, A101@CypA, H126@CypA, F113@CypA, M61@CypA, F60@CypA, L122@CypA (Table 5). Similar hydrophobic interactions stabilize *trans_180_*, but less persistently. Moreover, this conformation is stabilized by the R55@CypA…P4O@PEPT hydrophilic interaction. As for *cis_180_*, F60@CypA stabilizes also *trans_0_* (Table 5). The other CypA hydrophobic residues that stabilize the conformations at ψ∼±180° do not interact significantly in *trans_0_* (Table 5). At variance with any other minimum, the active site residues I57@CypA and W121@CypA stabilize solely *trans_0_*, i.e. the most stable conformation in water. The importance of W121 in the *trans* conformation was already reported in free energy calculations [15].

**Table 4:** Number of H-bonds of CypA residues with G3P4@PEPT along prolyl *cis/trans* isomerization.

CypA…G3P4@PEPT H-bonds	*trans_0_*	*Cis_0_*	*trans_180_*	*Cis_180_*	TS1	TS2	TS3	TS4
**Total**	0.1±0.0	0.6±0.0	1.6±0.0	1.3±0.0	0.1±0.0	1.9±0.0	1.5±0.0	1.1±0.0
**R55…P4O**	0±0	0±0	1.6±0.0	0±0	0±0	0±0	0±0	1.1±0.0
**N102N-H…P4O**	0±0	0.6±0.0	0±0	0±0	0±0	0±0	0±0	0±0
**N102N-H…G3O**	0±0	0±0	0±0	0.6±0.0	0±0	1.0±0.0	0.8±0.0	0±0
**N102O…G3N**	0±0	0±0	0±0	0.7±0.0	0±0	0.9±0	0.7±0.0	0±0
**W121…P4O**	0±0	0±0	0±0	0±0	0.1±0.0	0±0	0±0	0±0
**R148…P4O**	0.1±0.0	0±0	0±0	0±0	0±0	0±0	0±0	0±0

**Table 5:** Interface coordination number^a^ of CypA residues with G3P4@PEPT along prolyl *cis/trans* isomerization.

CypA Residues	trans_0_	cis_0_	trans_180_	cis_180_	TS1	TS2	TS3	TS4
**Total**	14.22±0.02	18.99±0.03	28.94±0.09	39.7±0.3	18.7±0.2	44.01±0.07	40.23±0.04	38.96±0.06
**Q63**	0.428±0.008	4.25±0.02	3.06±0.01	2.96±0.04	0.31±0.01	3.94±0.01	3.276±0.008	3.30±0.01
**A101**	0.107±0.002	3.83±0.02	2.73±0.02	3.35±0.04	0.189±0.008	4.94±0.01	3.829±0.007	4.47±0.01
**H126**	0.313±0.007	1.586±0.008	5.23±0.02	6.02±0.05	1.08±0.03	6.57±0.02	6.76±0.01	7.68±0.02
**F113**	0.470±0.008	1.97±0.01	6.77±0.04	11.4±0.1	0.66±0.03	12.79±0.04	11.77±0.02	2.95±0.01
**M61**	1.31±0.02	1.058±0.009	3.06±0.02	3.19±0.03	0.69±0.01	3.58±0.02	3.210±0.008	10.89±0.03
**F60**	3.84±0.04	0.410±0.004	1.35±0.03	5.49±0.04	4.4±0.1	4.98±0.02	4.17±0.02	1.38±0.02
**L122**	0.68±0.01	0.61±0.01	4.08±0.02	4.87±0.03	2.68±0.05	4.53±0.02	4.68±0.01	4.71±0.01
**I57**	3.19±0.03	0.412±0.006	0.883±0.008	0.597±0.006	1.21±0.03	0.577±0.002	0.542±0.001	0.611±0.002
**H54**	0.146±0.002	2.14±0.01	0.447±0.002	0.275±0.002	0.0589±0.0009	0.418±0.001	0.3447±0.0007	0.384±0.001
**A103**	0.111±0.002	2.34±0.01	0.504±0.005	0.463±0.005	0.150±0.004	0.612±0.002	0.631±0.001	1.49±0.01
**W121**	3.62±0.05	0.385±0.003	0.830±0.004	1.150±0.007	7.3±0.1	1.048±0.007	0.997±0.003	1.091±0.004

a Interface coordination number (IC) is defined as 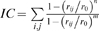, where IC is the index of G3P4@PEPT carbon atoms and j runs over the carbon atoms of CypA hydrophobic residues within 4Å of G3P4@PEPT.

N102 stabilizes *cis_0_*, as *cis_180_*, forming an H-bond to P4O. Several residues of CypA form hydrophobic interactions with *cis_0_*. Most of these residues stabilize also other minima (Q63, A101, F113). However, H54@CypA and A103@CypA stabilize only *cis_0_*, the minimum with the highest free energy.

The lowest free energy barrier is associated with the *trans_180_*→TS2→*cis_180_* (counterclockwise) path (Tables 2 and 3). This is also the only isomerization pathway catalyzed by the enzyme relative to PEPT-WAT (Tables 2 and 3). Preferential lowering of the counterclockwise N-terminal rotational free energy barrier is consistent with previous classic MD and free energy calculations [15],[57],[58].

Moreover, as previously noticed [58], N-terminal residue H1@PEPT is exposed to the solvent, while the C-terminal part is anchored to CypA active site (Table S6). TS2 (representative structure in Dataset S6) is stabilized by strong N102N…G3O@PEPT and N102O@CypA…G3N H-bonds (see Table 4) and by persistent hydrophobic interactions between the peptide and Q63, A101, H126, F113, F60 on CypA (see Table 5).

The enzyme does not decrease the barrier of the reverse pathway (*cis_180_*→TS2→*trans_180_*), as CypA stabilizes similarly *cis_180_* and TS2 (the latter is a bit more stabilized given the higher persistence of hydrophobic and hydrophilic interactions).

In addition, the H-bonds and hydrophobic contacts stabilize TS3 (representative structure in Dataset S7) and TS4 (representative structure in Dataset S8) along with their connected minima (Table 4, Table 2, and Figure 5). We further notice that the H-bonds in TS3 are the same as in TS2, although less persistent (Table 4).

TS1 (representative structure in Dataset S5) is not stabilized by H-bonds and interacts weakly with CypA hydrophobic residues (in particular, F60, L122 and W121, Table 4). Therefore the peptide in this conformation does not form a tight complex with the protein and it is exposed to the solvent (Figure 5). Thus, this isomerization pathway (*trans_0_*↔TS1↔*cis_0_*) is not too dissimilar from that in water and, indeed, the barriers for the two processes are practically identical (Table 2).

In each minimum and TS we identify large collective motions of the PEPT-CypA complex using PCA. We observe that significant modes involve almost the same residues as the motions found in Ref. [15](see Figure S6).

As for the peptide in water, we used other CV pairs (in addition to ζ, ψ) to describe the *cis*/*trans* isomerization. These include the pyramidalyzation as well as the number of H-bonds formed by P4N and residues of the peptide or of CypA. In addition, we included coordinates to take into account (i) the hydrophobic interaction between the peptide and the enzyme, and (ii) the interactions of R55 with both the peptide and the CypA active site (for a discussion of the other CV pairs see Figure S5, Text S1, and Table S7). All these calculations provide a consistent picture that leads us to conclude that the enzyme catalyzes only one pathway, the one from the most populated *trans* conformation, *trans_180_*, to the most stable minimum, *cis_180_*.

## Discussion

Our calculations produced the energetics of minima and TS of PEPT-WAT and PEPT-CypA. A critical discussion of the accuracy of the calculations is necessary to proceed with possible insights on the enzyme working mechanism. The source of errors in a free energy calculations are basically of two types [59].

The first is caused by a non-complete sampling (statistical error): our estimated statistical error turns out to be relatively small, less than 1.5 kcal/mol (see Text S1).

The second is caused by the force field. This is expected to be particularly relevant for the TSs, because P4N passes from almost sp^2^ hybridization (resonance of prolyl-peptidyl bond) in minima to sp^3^ at the TSs (the so-called pyramidalization). Notice that since the Amber99 force-field is not designed for having all bonds fixed [29], the isomerization barriers might have been overestimated. However, we expect this effect to bias all barriers in the same direction, so that our conclusions on free-energy differences should not be affected by this choice.

To estimate the error due to the force field, quantum chemical calculations are compared with force field based ones on model systems. Previous work has shown that DFT-B3LYP with 6–31G(d) basis set provide similar results as more expensive (albeit usually more accurate) MP2 calculations on the N-acetyl-N-N′-methyl proline amide [60]. Therefore, here we limit ourselves to a DFT-B3LYP/6–31G(d) calculation on the N-acetyl proline methylamide system, in vacuum, at 0 K, and compare our results with AMBER calculations.

We find small differences for the minima (of the same magnitude of the statistical error, 1.5 kcal/mol, see Text S1). Instead, the differences are 4 kcal/mol or lower for the barriers. We further notice that in three of the reversible pathways (*cis_0_*↔TS1↔*trans_0_*; *cis_180_*↔TS2↔*trans_180_*; *cis_180_*↔TS4↔*trans_180_*), DFT barriers are higher than force field ones, while QM barriers of *cis_0_*↔TS3↔*trans_0_* are slightly lower than the force-field ones (Table S4).

We conclude that our calculations may provide a relatively accurate estimate of the Boltzmann populations of *cis* and *trans* free energy minima. In addition, as the errors associated to the barriers are likely to be similar for all pathways, our calculations can be used for qualitative comparisons of the free energy barriers, i.e. to identify the lowest free energy pathways.

### Population of Free Energy Minima

In water, *trans_0_* is by far the most populated specie (Table 2). The same result for the potential energy has been obtained with DFT (see Text S1) gas phase calculations on N-acetylproline methylamide.

The protein environment changes dramatically the populations of the proline conformers, stabilizing *cis* with respect to *trans*, as previously reported by CypA-SUC-Ala-Phe-Pro-Phe-pNA complex [17] and CypA^R55A^-CA [12]. However, our study provides a quantitative estimate of the populations of conformers (Table 2): in presence of the enzyme, three conformers (*trans_0_*, *trans_180_* and *cis_180_*) are significantly populated. The most populated one corresponds to *cis_180_*, that is exactly the most unfavorable state in aqueous environment. This finding has never been reported in literature.

### Qualitative Comparison of Free Energy Barriers

CypA accelerates only one interconversion, namely the one from *trans_180_* to TS2 to *cis_180_* (Table 3), as already suggested by previous calculations [15]): our calculated free energy barrier decreases by 4 kcal/mol with respect to the peptide in water. Indeed, as well as in the other pathways, almost no effect is found on the reverse pathway (*cis_180_*→TS2→*trans_180_*, 1 kcal/mol, i.e. within the error of the calculations), again in agreement with Refs. [14],[15] (Table 3). The *trans_180_*→TS2→*cis_180_* is also associated with the lowest free energy barrier. We conclude that this pathway is the most likely in the enzyme, although we cannot establish with high accuracy the free energy barrier of the enzymatic reaction.

**Figure 6:**
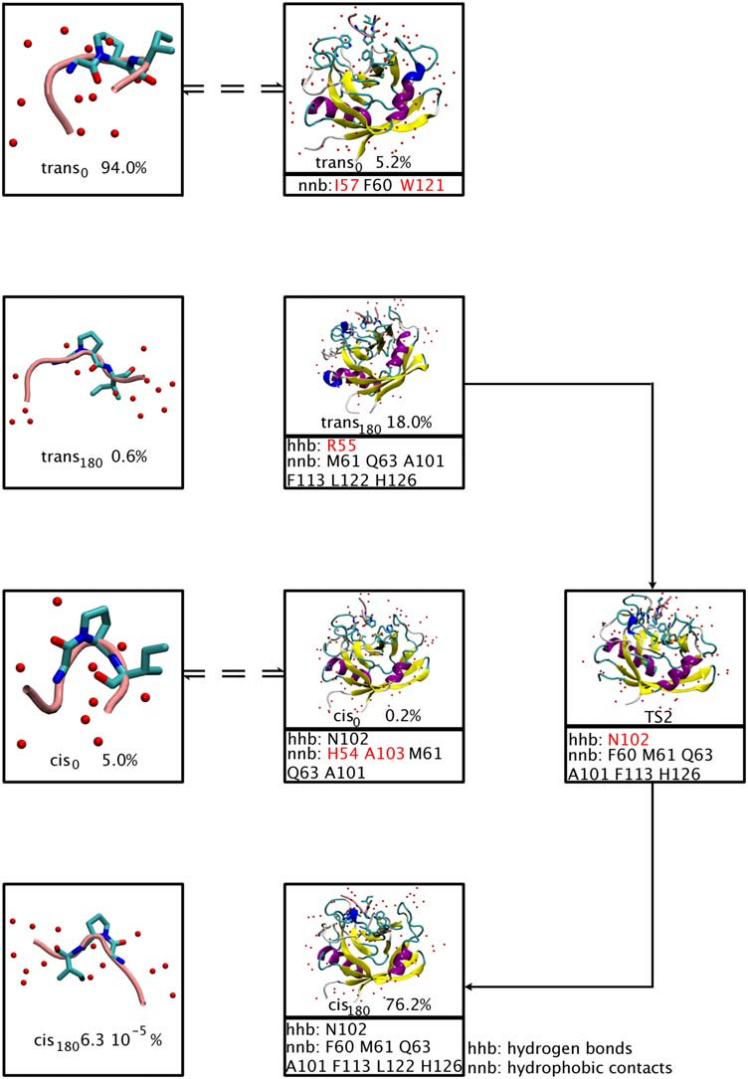
Proposed mechanism of action for CypA. CypA sequesters the most abundant conformation in water, *trans_0_*, that is rapidly interconverted into the most abundant conformer *trans_180_*. Then, CypA catalyzes the isomerization of the peptide along TS2, producing the mostly populated minimum, *cis_180_*. The peptide most probably detaches in the *cis_180_* conformation. This readily interconverts to *cis_0_* once the peptide is in aqueous solution. CypA residues that form important H-bonds (hhb) and hydrophobic interactions (nnb) with the G3P4 moiety are shown. Residues with almost exclusive relevance to each conformation are highlighted in red.

Based on our calculation, we propose the following mechanism (Figure 6):

1. The most abundant conformation of the peptide in water is, as it is well known [12], *trans* conformation. Specifically, in our system it is *trans_0_*.
2. CypA sequesters *trans_0_* and rapidly interconverts it into *trans_180_*, because the population of the latter is significantly greater in the protein complex. Notice that the process is much faster than the conversion to *cis_180_* or to *cis_0_*, as the activation free energy associated with them is one order of magnitude smaller.
3. The enzyme catalyzes the isomerization to the most populated minimum, *cis_180_*, (*trans_180_*→TS2→*cis_180_* pathway). This minimum is strongly stabilized by the same PEPT-CypA interactions as those observed in the most stabilized transition state (TS2), but less persistently (Table 4). Notice that the reverse interconversion from *cis_180_* to *trans_180_* is not likely because: (i) the free energy barrier associated with it is larger (indeed, the *trans_180_*→TS2→*cis_180_* pathway is the *only* pathway catalyzed by the enzyme) and (ii) the population of *trans_180_* is a few times smaller than *cis_180_* (Table 2). This contrasts with isomerization in water solution, where the population of the most stable *trans* conformation (*trans_0_*) is several orders of magnitude higher than the one of the preponderant *cis* (*cis_0_*).
4. The enzyme can now either detach *cis_180_* or interconvert it to *cis_0_*. However, only in the latter case H54 does interact with the peptide, consistently with the experimental fact that the H54Q mutation decreases the enzymatic activity; the k_cat_/K_m_ is reduced to the 15% of its wild-type value [20]. Notice that all the enzymatic activity measures, for mutants of polymorphism, do not provide information on k_cat_ or K_m_ alone but only on their ratio. Therefore one cannot conclude whether a mutation affects either k_cat_ or K_m_ or both. Thus, it is plausible to assume that the enzyme interconverts *cis_180_* to *cis_0_*. This assumption may be validated against further molecular biology experiments: in particular, it would be highly useful to measure the activity of A103X mutants (X = polar or charged residues), because A103 stabilizes only *cis_0_*.
5. *cis_0_* is released.

Our mechanism provides a first rationale for mutational data which have not been explained so far. The decreased k_cat_/K_m_ values (Table S1) of the W121A mutant [20] and the I57V variant [19] cannot be explained in terms of loss of TS stabilization. In our mechanism, these two mutations are likely to affect substrate stabilization in the *trans_0_* conformation, reducing CypA ability to capture the substrate. In addition, the dramatic decrease of enzyme efficiency in the R55A mutant (0.10% residual activity [20]), whose effect on TS stabilization is controversial, may be, at least in part, a consequence of the reduced population of *trans_180_*, the reactant of the CypA catalyzed pathway (*trans_180_*→TS2→*cis_180_*).

Next, we analyze the effect of several mutations and polymorphism, so far ascribed only to TS stabilization [14],[15],[18], as also found here, that might also have an impact on ground state populations.

Indeed, reduced k_cat_/K_m_ values on enzymes where N102 exchanges to T, H and R residues [19] may be not only due to destabilization of the TS, but also of the *cis_180_* conformation. Similarly, some CypA mutations also decrease k_cat_/K_m_: (i) F60A [20] may destabilize TS2, *trans_0_* and *cis_180_*; (ii) F113A and H126Q [20] may affect all the species along the *trans_180_*→TS2→*cis_180_* pathway (Table S1).

*cis_0_*, proposed to be the most probable final step in our mechanism based on H54Q mutant, is stabilized exclusively by interactions of H54 and A103 with G3P4.

We conclude that our mechanism is consistent with all the mutational and polymorphism data and provides a structural basis for most of them.

## Supporting Information

Dataset S1 Representative structure of trans0 conformation (center of the lower free energy cluster within trans0 region) in PDB format.
(0.21 MB TXT)

Dataset S2 Representative structure of trans180 conformation (center of the lower free energy cluster within trans180 region) in PDB format.
(0.21 MB TXT)

Dataset S3 Representative structure of cis0 conformation (center of the lower free energy cluster within cis0 region) in PDB format.
(0.21 MB TXT)

Dataset S4 Representative structure of cis180 conformation (center of the lower free energy cluster within cis180 region) in PDB format.
(0.21 MB TXT)

Dataset S5 Representative structure of TS1 conformation (center of the lower free energy cluster within TS1 region) in PDB format.
(0.21 MB TXT)

Dataset S6 Representative structure of TS2 conformation (center of the lower free energy cluster within TS2 region) in PDB format.
(0.21 MB TXT)

Dataset S7 Representative structure of TS3 conformation (center of the lower free energy cluster within TS3 region) in PDB format.
(0.21 MB TXT)

Dataset S8 Representative structure of TS4 conformation (center of the lower free energy cluster within TS4 region) in PDB format.
(0.21 MB TXT)

Figure S1 Left: non catalyzed /HAGPIA/ peptide cis/trans prolyl isomerization: free energy (kcal/mol) as a function of the dihedral angles ζ and ψ (in degrees, showed in the inset). The plot is divided in transition and minimum regions. Center: each transition and minimum region contains i clusters. Right: the structures within each cluster i are analyzed measuring χ^2^ and NHB parameters.
(1.15 MB TIF)

Figure S2 Non catalyzed /HAGPIA/ peptide cis/trans prolyl isomerization: free energy (kcal/mol) and enthalpy (kcal/mol) as a function of the dihedral angle ζ (in degrees) obtained by integrating out the variable p. The entropic contribution is given by the difference between the two curves. The isomerization process is mainly enthalpy-driven.
(0.15 MB TIF)

Figure S3 (Top) Up, planar and down proline ring puckering conformations. Puckering is defined based on the value of the χ^2^ dihedral angle (Cα-C β-C γ-C δ): up-puckering for χ^2^>10°, planar puckering for −10°<χ^2^<10°, down-puckering for χ^2^<−10°. (Bottom) χ^2^ distribution within clusters classified as up, down and planar puckering. χ^2^ has a bimodal distribution in all clusters with a largest maximum at χ^2^ = +40° for up clusters, at χ^2^ = −40° for down clusters and with two even peaks at −40° and +40° for planar clusters.
(0.27 MB TIF)

Figure S4 (A) Non catalyzed /HAGPIA/ peptide cis/trans prolyl isomerization: free energy (kcal/mol) as a function of the dihedral angles ζ (in degrees, showed in the inset) and the pyramidalization p. (B) Non catalyzed /HAGPIA/ peptide cis/trans prolyl isomerization: free energy (kcal/mol) as a function of the dihedral angles ζ (in degrees, showed in the inset) and the coordination of P4N with peptide H-bond donors (s1). (C) Non catalyzed /HAGPIA/ peptide cis/trans prolyl isomerization: free energy (kcal/mol) as a function of the dihedral angles ζ (in degrees, showed in the inset) and the coordination of P4N with all water molecules (s2). (D) Non catalyzed /HAGPIA/ peptide cis/trans prolyl isomerization: free energy (kcal/mol) as a function of the pyramidalization (p) and the coordination of P4N with peptide H-bond donors (s1). (E) Non catalyzed /HAGPIA/ peptide cis/trans prolyl isomerization: free energy (kcal/mol) as a function of the pyramidalization (p) and the coordination of P4N with all water molecules (s2).
(2.08 MB TIF)

Figure S5 (A) Enzyme catalyzed /HAGPIA/ peptide cis/trans prolyl isomerization: free energy (kcal/mol) as a function of the dihedral angles ζ and the pyramidalization p. (B) Enzyme catalyzed /HAGPIA/ peptide cis/trans prolyl isomerization: free energy (kcal/mol) as a function of the dihedral angles ζ and the coordination of P4N with peptide and enzyme H-bond donors (s1). (C) Enzyme catalyzed /HAGPIA/ peptide cis/trans prolyl isomerization: free energy (kcal/mol) as a function of the dihedral angles ζ and the hydrophobic coordination of G3P4 with CypA (s3). (D) Enzyme catalyzed /HAGPIA/ peptide cis/trans prolyl isomerization: free energy (kcal/mol) as a function of the dihedral angles ζ and the hydrophobic coordination of pepide C- and N- termini (H1, A2, I5, A6) with CypA (s4). (E) Enzyme catalyzed /HAGPIA/ peptide cis/trans prolyl isomerization: free energy (kcal/mol) as a function of the dihedral angles ζ and the hydrophobic coordination of peptide C-term (I5A6) with L89 and S90 (s5). (F) Enzyme catalyzed /HAGPIA/ peptide cis/trans prolyl isomerization: free energy (kcal/mol) as a function of the dihedral angles ζ and the coordination of R55 with P4N and protein H-bond donors (s6). (G) Enzyme catalyzed /HAGPIA/ peptide cis/trans prolyl isomerization: free energy (kcal/mol) as a function of the pyramidalization p and the coordination of P4N with peptide H-bond donors (s1).
(1.85 MB TIF)

Figure S6 Normal modes for fluctuations observed on each minimum and the catalytic TS (TS2) of the enzyme catalyzed /HAGPIA/ isomerization. The modes are calculated from principal component analysis (PCA). Relevant modes (high eigenvalue) that are similar to fluctuations reported previously [15] are displayed.
(2.01 MB TIF)

Table S1 Isomerase activity of several wild type cyclophilins and CypA mutants.
(0.04 MB DOC)

Table S2 Collective variables (CVs) and parameters used in bias-exchange metadynamics calculations
(0.08 MB DOC)

Table S3 Structural determinants and energetics of PEPT: averaged P4N….I5H-N H-bond number (NHB) and puckering (χ^2^ angle, Figure 2) within each region of the F(ζ, ψ) free energy profile.
(0.03 MB DOC)

Table S4 Potential energy barriers (kcal/mol) for N-Acetylproline methylamide isomerization at the B3LYP/6-31G(d) level of theory and with the Amber99 force field.
(0.04 MB DOC)

Table S5 ζ and ψ (Figure 2) values used in N-Acetylproline methylamide partial optimization at the B3LYP/6-31G(d) level of theory and with the Amber99 force field. Parameters of the NPro…H-Ni+1 hydrogen bond and potential energy of each structure are included.
(0.05 MB DOC)

Table S6 Number of H-bonds between PEPT-CypA complex and water molecules
(0.03 MB DOC)

Table S7 Average distances between G3@PEPT and L98S99@CypA
(0.03 MB DOC)

Text S1 Clustering, error evaluation, puckering of peptide in water and analysis of secondary free energy profiles obtained in the bias exchange metadynamics simulations.
(0.10 MB DOC)

## References

[1] Fischer S, Dunbrack RL, Karplus M (1994). Cis-trans imide isomerization of the proline dipeptide.. J Am Chem Soc.

[2] Hodel A, Rice LM, Simonson T, Fox RO, Brunger AT (1995). Proline cis-trans isomerization in Staphylococcal nuclease: multi-substrate free energy perturbation calculations.. Protein Sci.

[3] Harrar Y, Bellini C, Faure JD (2001). FKBPs: at the crossroads of folding and transduction.. Trends Plant Sci.

[4] Wang P, Heitman J (2005). The cyclophilins.. Genome Biol.

[5] Lummis SCR, Beene DL, Lee LW, Lester HA, Broadhurst RW (2005). Cis-trans isomerization at a proline opens the pore of a neurotransmitter-gated ion channel.. Nature.

[6] Wulf G, Finn G, Suizu F, Lu KP (2005). Phosphorylation-specific prolyl isomerization: is there an underlying theme?. Nat Cell Biol.

[7] Scarlata S, Carter C (2003). Role of HIV-1 Gag domains in virial assembly.. Biochim Biophys Acta.

[8] Göthel SF, Marahiel MA (1999). Peptidyl-prolyl cis-trans isomerases, a superfamily of ubiquitous folding catalysts.. Cell Mol Life Sci.

[9] Pastorino L, Sun A, Lu PJ, Zhou XZ, Balastik M (2006). The prolyl isomerase Pin1 regulates amyloid precursor protein processing and amyloid-β production.. Nature.

[10] Finkelstein AV, Ptitsyn OB (2002). Lecture 2.. Protein Physics: A Course of Lectures.

[11] van Holde KE, Jonson WC, Ho PS (1998). Biological macromolecules.. Principles of Physical Biochemistry.

[12] Bosco DA, Eisenmesser EZ, Pochapsky S, Sundquist WI, Kern D (2002). Catalysis of cis/trans isomerization in native HIV-1 capsid by human cyclophilin A.. Proc Natl Acad Sci U S A.

[13] Howard BR, Vajdos FF, Li S, Sundquist WI, Hill CP (2003). Structural insights into the catalytic mechanism of cyclophilin A.. Nat Struct Biol.

[14] Li G, Cui Q (2003). What is so special about Arg 55 in the catalysis of cyclophilin A? insights from hybrid QM/MM simulations.. J Am Chem Soc.

[15] Agarwal PK, Geist A, Gorin A (2004). Protein dynamics and enzymatic catalysis: investigating the peptidyl-prolyl cis-trans isomerization activity of cyclophilin A.. Biochemistry.

[16] Kofron JL, Kuzmic P, Kishore V, Colón-Bonilla E, Rich DH (1991). Determination of kinetic constants for peptidyl prolyl cis-trans isomerases by an improved spectrophotometric assay.. Biochemistry.

[17] Kern D, Kern G, Scherer G, Fischer G, Drakenberg T (1995). Kinetic analysis of cyclophilin-catalyzed prolyl cis/trans isomerization by dynamic NMR spectroscopy.. Biochemistry.

[18] Hur S, Bruice TC (2002). The mechanism of cis-trans isomerization of prolyl peptides by cyclophilin.. J Am Chem Soc.

[19] Fanghänel J, Fischer G (2004). Insights into the catalytic mechanism of peptidyl prolyl cis/trans isomerases.. Front Biosci.

[20] Zydowsky LD, Etzkorn FA, Chang HY, Ferguson SB, Stolz LA (1992). Active site mutants of human cyclophilin A separate peptidyl-prolyl isomerase activity from cyclosporin A binding and calcineurin inhibition.. Protein Sci.

[21] Eisenmesser EZ, Bosco DA, Akke M, Kern D (2002). Enzyme dynamics during catalysis.. Science.

[22] Agarwal PK (2004). Cis/trans isomerization in HIV-1 capsid protein catalyzed by cyclophilin A: insights from computational and theoretical studies.. Proteins.

[23] Agarwal PK (2005). Role of protein dynamics in reaction rate enhancement by enzymes.. J Am Chem Soc.

[24] Zhao Y, Ke H (1996). Crystal structure implies that cyclophilin predominantly catalyzes the trans to cis isomerization.. Biochemistry.

[25] Fischer S, Dunbrack RL, Karplus M (1994). Cis-trans imide isomerization of the proline dipeptide.. J Am Chem Soc.

[26] Laio A, Parrinello M (2002). Escaping free-energy minima.. Proc Natl Acad Sci U S A.

[27] Piana S, Laio A (2007). A bias-exchange approach to protein folding.. J Phys Chem B.

[28] Fiorin G, Pastore A, Carloni P, Parrinello M (2006). Using metadynamics to understand the mechanism of calmodulin/target recognition at atomic detail.. Biophys J.

[29] Wang JM, Cieplak P, Kollman PA (2000). How well does a restrained electrostatic potential (RESP) model perform in calculating conformational energies of organic and biological molecules?. J Comput Chem.

[30] Hornak V, Abel R, Okur A, Strockbine B, Roitberg A (2006). Comparison of multiple Amber force fields and development of improved protein backbone parameters.. Proteins.

[31] Bashford D, Karplus M (1990). pK_a_'s of ionizable groups in proteins - Atomic detail from a continuum electrostatic model.. Biochemistry.

[32] Gordon JC, Myers JB, Folta T, Shoja V, Heath LS (2005). H++: a server for estimating pK_a_s and adding missing hydrogens to macromolecules.. Nucleic Acids Res.

[33] Kelly BN, Kyere S, Kinde I, Tang C, Howard BR (2007). Structure of the antiviral assembly inhibitor CAP-1 complex with the HIV-1 CA protein.. J Mol Biol.

[34] Mahoney MW, Jorgensen WL (2000). A five-site model for liquid water and the reproduction of the density anomaly by rigid, nonpolarizable potential functions.. J Chem Phys.

[35] Feller SE, Zhang YH, Pastor RW, Brooks BR (1995). Constant-pressure molecular-dynamics simulation: the Langevin piston method.. J Chem Phys.

[36] Paterlini MG, Ferguson DM (1998). Constant temperature simulations using the Langevin equation with velocity Verlet integration.. Chem Phys.

[37] Essmann U, Perera L, Berkowitz ML, Darden T, Lee H (1995). A smooth particle mesh Ewald method.. J Chem Phys.

[38] Phillips JC, Braun R, Wang W, Gumbart J, Tajkhorshid E (2005). Scalable molecular dynamics with NAMD.. J Comput Chem.

[39] Metropolis N, Rosenbluth AW, Rosenbluth MN, Teller AH, Teller E (1953). Equation of state calculations by fast computing machines.. J Chem Phys.

[40] Daura X, Gademann K, Jaun B, Seebach D, van Gunsteren WF (1999). Peptide folding: when simulation meets experiment.. Angew Chem Int Ed Engl.

[41] Marinelli F, Pietrucci F, Piana S, Laio A (2008). A kinetic model of Trp-cage folding from multiple biased molecular dynamics simulations.. Submitted.

[42] Evans DJ, Holian BL (1985). The Nose-Hoover thermostat.. J Chem Phys.

[43] Hess B, Bekker H, Berendsen HJC, Fraaije JGEM (1997). LINCS: a linear constraint solver for molecular simulations.. J Comput Chem.

[44] Lindahl E, Hess B, van der Spoel D (2001). GROMACS 3.0: a package for molecular simulation and trajectory analysis.. J Mol Model.

[45] Berendsen HJC, Vanderspoel D, Vandrunen R (1995). GROMACS: a message-passing parallel molecular dynamics implementation.. Comput Phys Commun.

[46] Piana S, Laio A, Marinelli F, Van Troys M, Bourry D (2008). Predicting the effect of a point mutation on a protein fold: the villin and advillin headpieces and their Pro62Ala mutants.. J Mol Biol.

[47] Ho BK, Coutsias EA, Seok C, Dill KA (2005). The flexibility in the proline ring couples to the protein backbone.. Protein Sci.

[48] Garcia AE (1992). Large-amplitude nonlinear motions in proteins.. Phys Rev Lett.

[49] Amadei A, Linssen AB, Berendsen HJ (1993). Essential dynamics of proteins.. Proteins.

[50] Lee CT, Yang WT, Parr RG (1988). Development of the Colle-Salvetti correlation-energy formula into a functional of the electron density.. Phys Rev B.

[51] Becke AD (1993). Density-functional thermochemistry III. The role of exact exchange.. J Chem Phys.

[52] Vosko SH, Wilk L, Nusair M (1980). Accurate spin-dependent electron liquid correlation energies for local spin density calculations: a critical analysis.. Can J Phys.

[53] Stephens PJ, Devlin FJ, Chabalowski CF, Frisch MJ (1994). Ab initio calculation of vibrational absorption and circular dichroism spectra using density functional force fields.. J Phys Chem.

[54] Frisch MJ (1998). GImage 98.

[55] Stein RL (1993). Mechanism of enzymatic and nonenzymatic prolyl cis-trans isomerization.. Adv Protein Chem.

[56] Eberhardt ES, Loh SN, Hinck AP, Raines RT (1992). Solvent effects on the energetics of prolyl peptide bond isomerization.. J Am Chem Soc.

[57] Trzesniak D, van Gunsteren WF (2006). Catalytic mechanism of cyclophilin as observed in molecular dynamics simulations: pathway prediction and reconciliation of X-ray crystallographic and NMR solution data.. Protein Sci.

[58] Mark P, Nilsson L (2007). A molecular dynamics study of cyclophilin A free and in complex with the Ala-Pro dipeptide.. Eur Biophys J.

[59] McDowell SE, Špačková N, Šponer J, Walter NG (2007). Molecular dynamics simulations of RNA: an in silico single molecule approach.. Biopolymers.

[60] Jhon JS, Kang YK (1999). Imide Cis-Trans isomerization of *N*-Acetyl-*N*′-methyl proline amide and solvent effects.. J Phys Chem A.

