# Supplementary figures and images for: Mechanism of Action of Cyclophilin A Explored by Metadynamics Simulations
Source: PLoS Comput Biol. 2009 Mar 13;5(3):e1000309. doi: 10.1371/journal.pcbi.1000309 (PMC2643488; doi:10.1371/journal.pcbi.1000309)

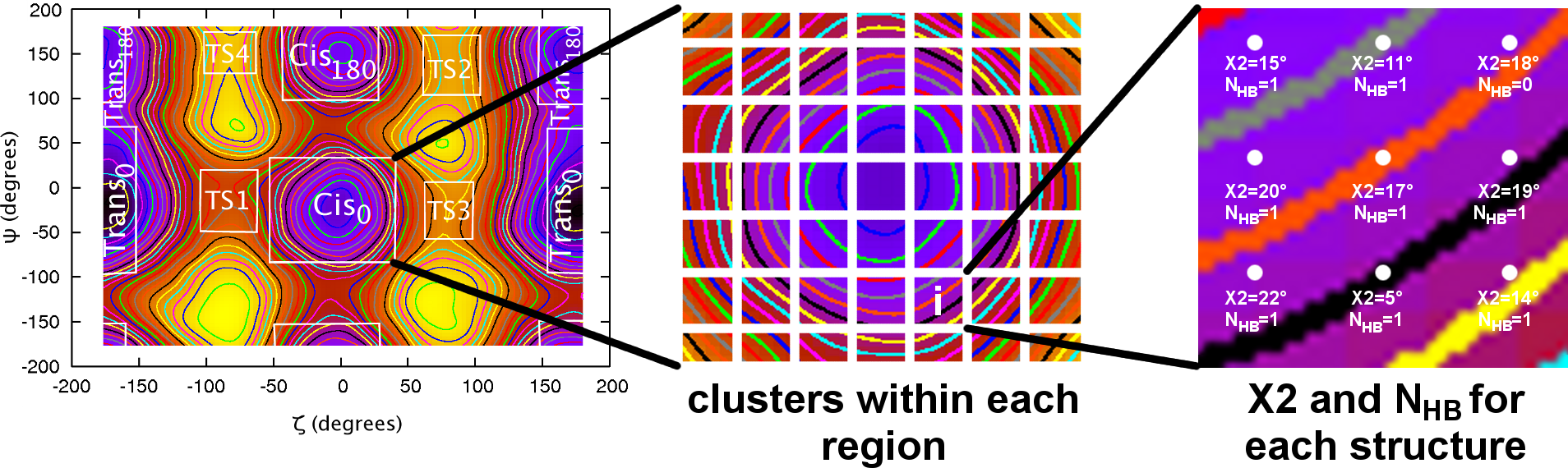

Supplement: Figure S1 — Left: non catalyzed /HAGPIA/ peptide cis/trans prolyl isomerization: free energy (kcal/mol) as a function of the dihedral angles ζ and ψ (in degrees, showed in the inset). The plot is divided in transition and minimum regions. Center: each transition and minimum region contains i clusters. Right: the structures within each cluster i are analyzed measuring χ2 and NHB parameters. (1.15 MB TIF) [file pcbi.1000309.s009.tif]

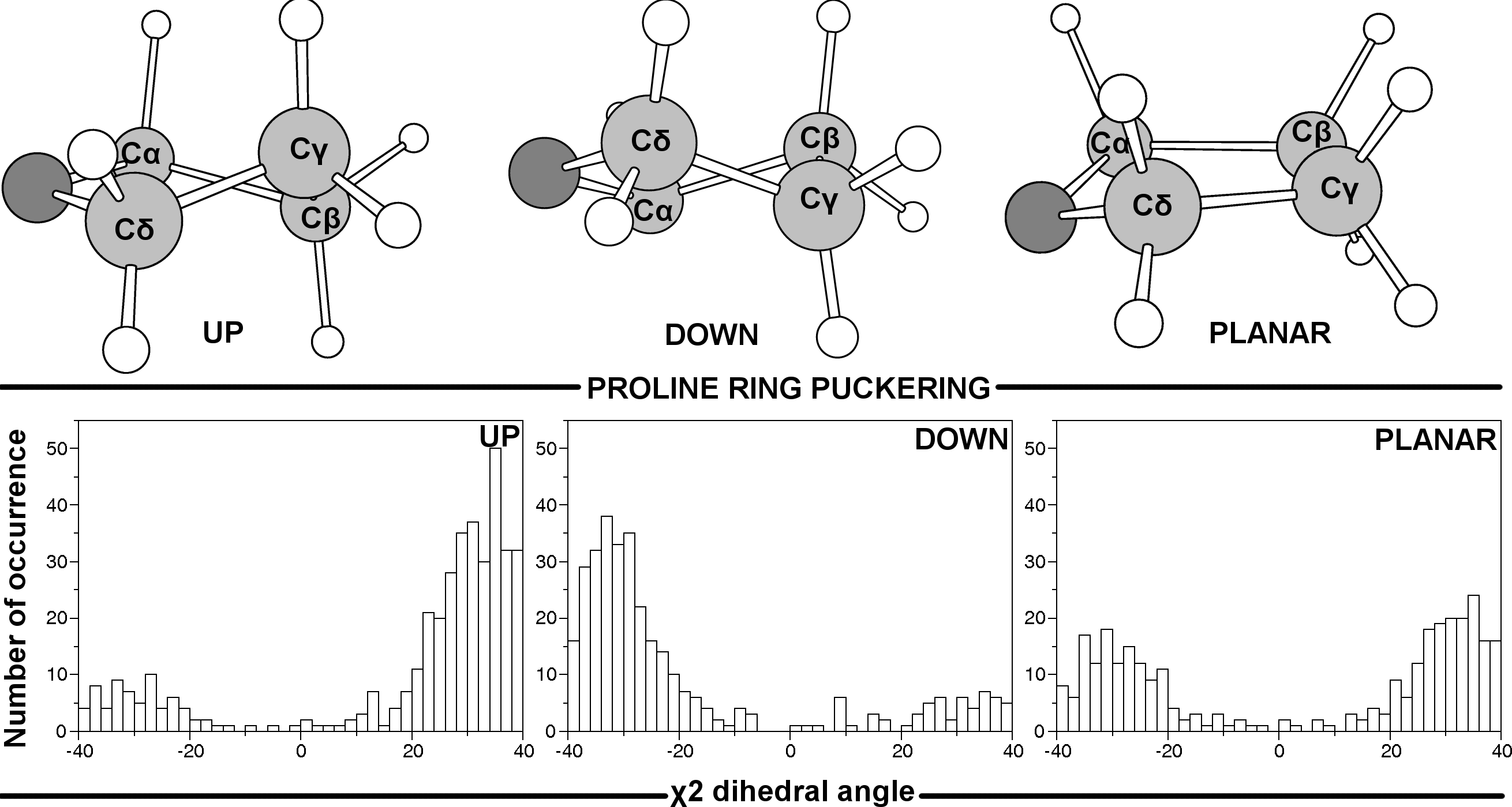

Supplement: Figure S3 — (Top) Up, planar and down proline ring puckering conformations. Puckering is defined based on the value of the χ2 dihedral angle (Cα-C β-C γ-C δ): up-puckering for χ2>10°, planar puckering for −10°<χ2<10°, down-puckering for χ2<−10°. (Bottom) χ2 distribution within clusters classified as up, down and planar puckering. χ2 has a bimodal distribution in all clusters with a largest maximum at χ2 = +40° for up clusters, at χ2 = −40° for down clusters and with two even peaks at −40° and +40° for planar clusters. (0.27 MB TIF) [file pcbi.1000309.s011.tif]

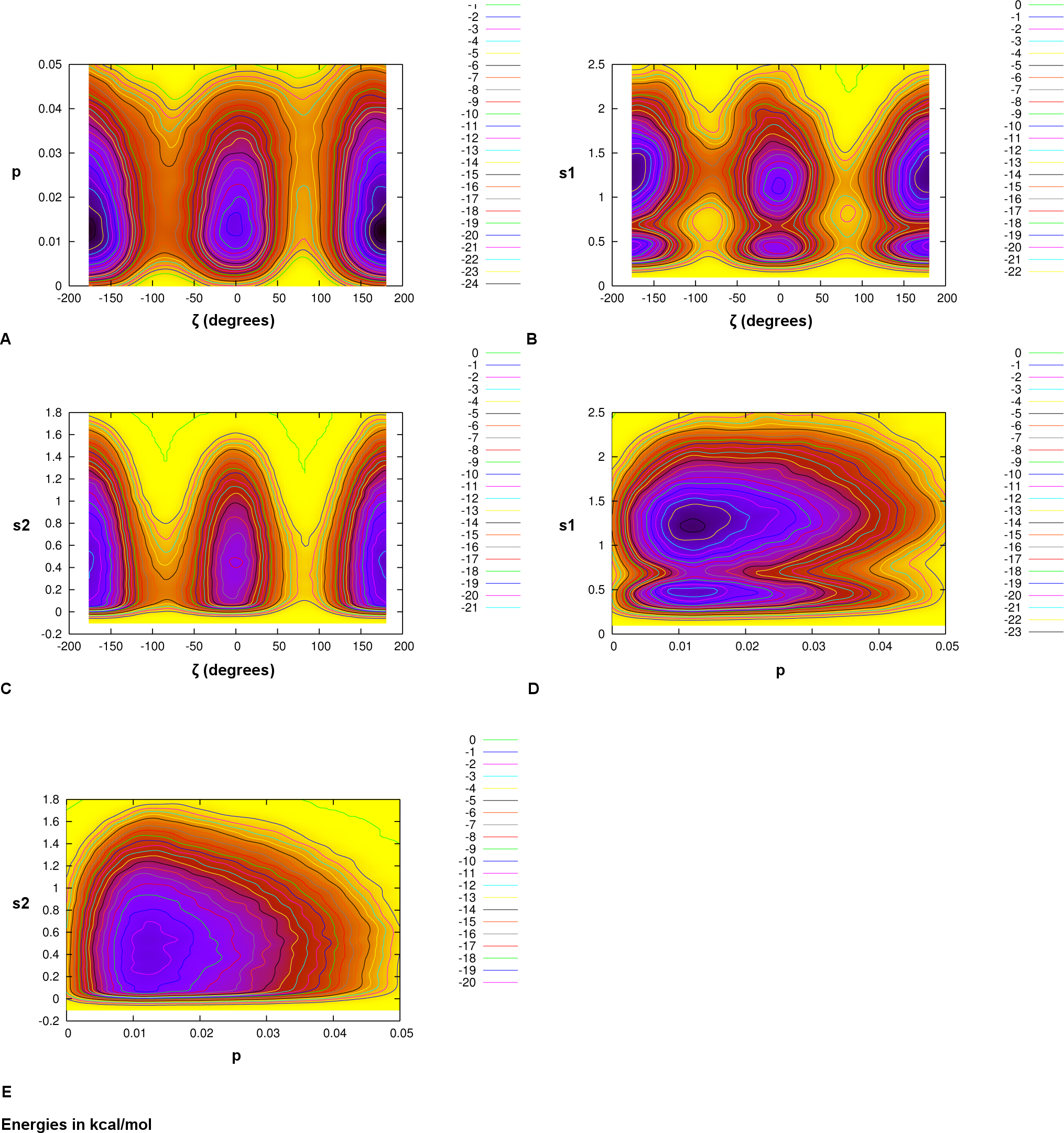

Supplement: Figure S4 — (A) Non catalyzed /HAGPIA/ peptide cis/trans prolyl isomerization: free energy (kcal/mol) as a function of the dihedral angles ζ (in degrees, showed in the inset) and the pyramidalization p. (B) Non catalyzed /HAGPIA/ peptide cis/trans prolyl isomerization: free energy (kcal/mol) as a function of the dihedral angles ζ (in degrees, showed in the inset) and the coordination of P4N with peptide H-bond donors (s1). (C) Non catalyzed /HAGPIA/ peptide cis/trans prolyl isomerization: free energy (kcal/mol) as a function of the dihedral angles ζ (in degrees, showed in the inset) and the coordination of P4N with all water molecules (s2). (D) Non catalyzed /HAGPIA/ peptide cis/trans prolyl isomerization: free energy (kcal/mol) as a function of the pyramidalization (p) and the coordination of P4N with peptide H-bond donors (s1). (E) Non catalyzed /HAGPIA/ peptide cis/trans prolyl isomerization: free energy (kcal/mol) as a function of the pyramidalization (p) and the coordination of P4N with all water molecules (s2). (2.08 MB TIF) [file pcbi.1000309.s012.tif]

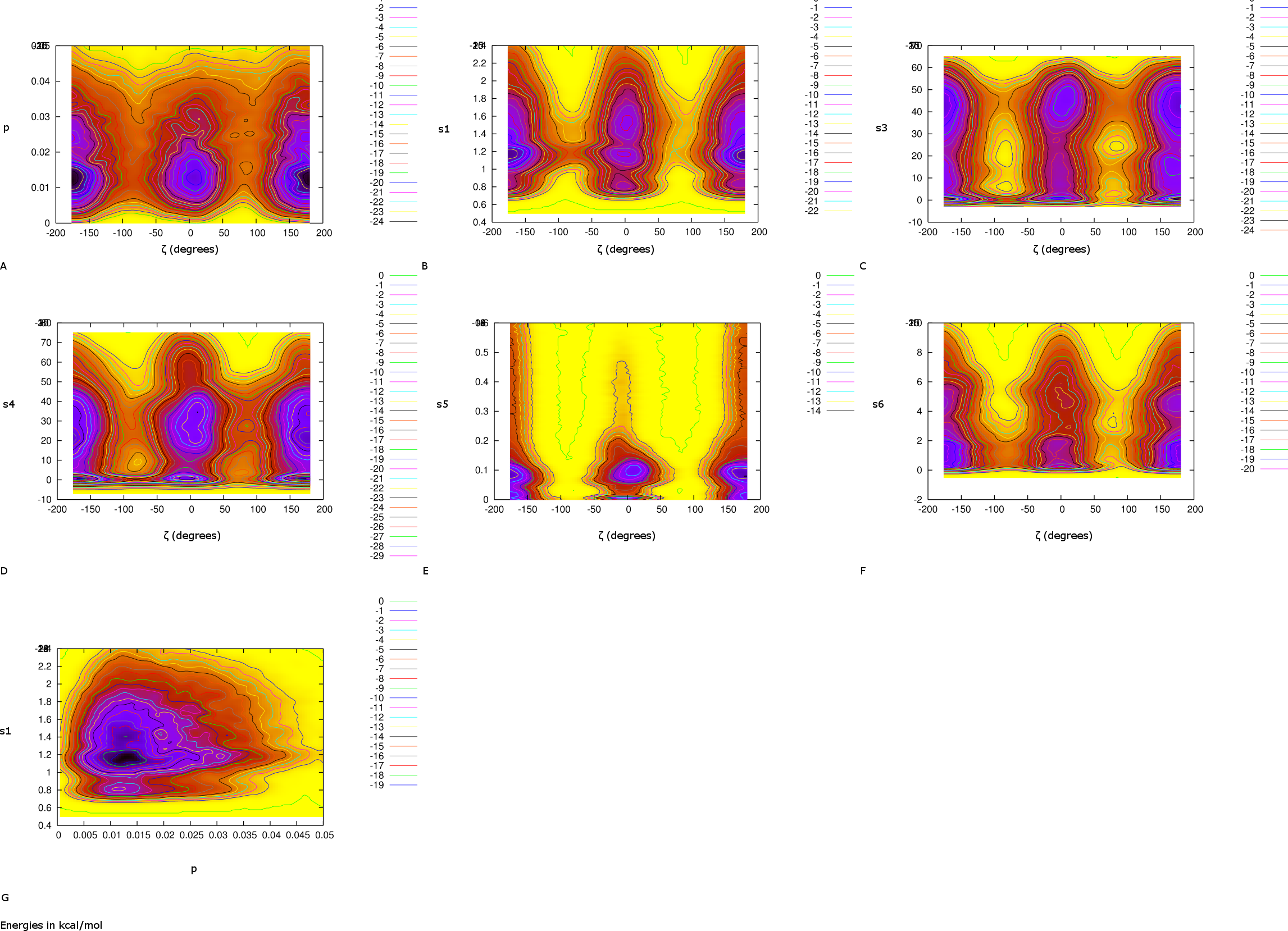

Supplement: Figure S5 — (A) Enzyme catalyzed /HAGPIA/ peptide cis/trans prolyl isomerization: free energy (kcal/mol) as a function of the dihedral angles ζ and the pyramidalization p. (B) Enzyme catalyzed /HAGPIA/ peptide cis/trans prolyl isomerization: free energy (kcal/mol) as a function of the dihedral angles ζ and the coordination of P4N with peptide and enzyme H-bond donors (s1). (C) Enzyme catalyzed /HAGPIA/ peptide cis/trans prolyl isomerization: free energy (kcal/mol) as a function of the dihedral angles ζ and the hydrophobic coordination of G3P4 with CypA (s3). (D) Enzyme catalyzed /HAGPIA/ peptide cis/trans prolyl isomerization: free energy (kcal/mol) as a function of the dihedral angles ζ and the hydrophobic coordination of pepide C- and N- termini (H1, A2, I5, A6) with CypA (s4). (E) Enzyme catalyzed /HAGPIA/ peptide cis/trans prolyl isomerization: free energy (kcal/mol) as a function of the dihedral angles ζ and the hydrophobic coordination of peptide C-term (I5A6) with L89 and S90 (s5). (F) Enzyme catalyzed /HAGPIA/ peptide cis/trans prolyl isomerization: free energy (kcal/mol) as a function of the dihedral angles ζ and the coordination of R55 with P4N and protein H-bond donors (s6). (G) Enzyme catalyzed /HAGPIA/ peptide cis/trans prolyl isomerization: free energy (kcal/mol) as a function of the pyramidalization p and the coordination of P4N with peptide H-bond donors (s1). (1.85 MB TIF) [file pcbi.1000309.s013.tif]

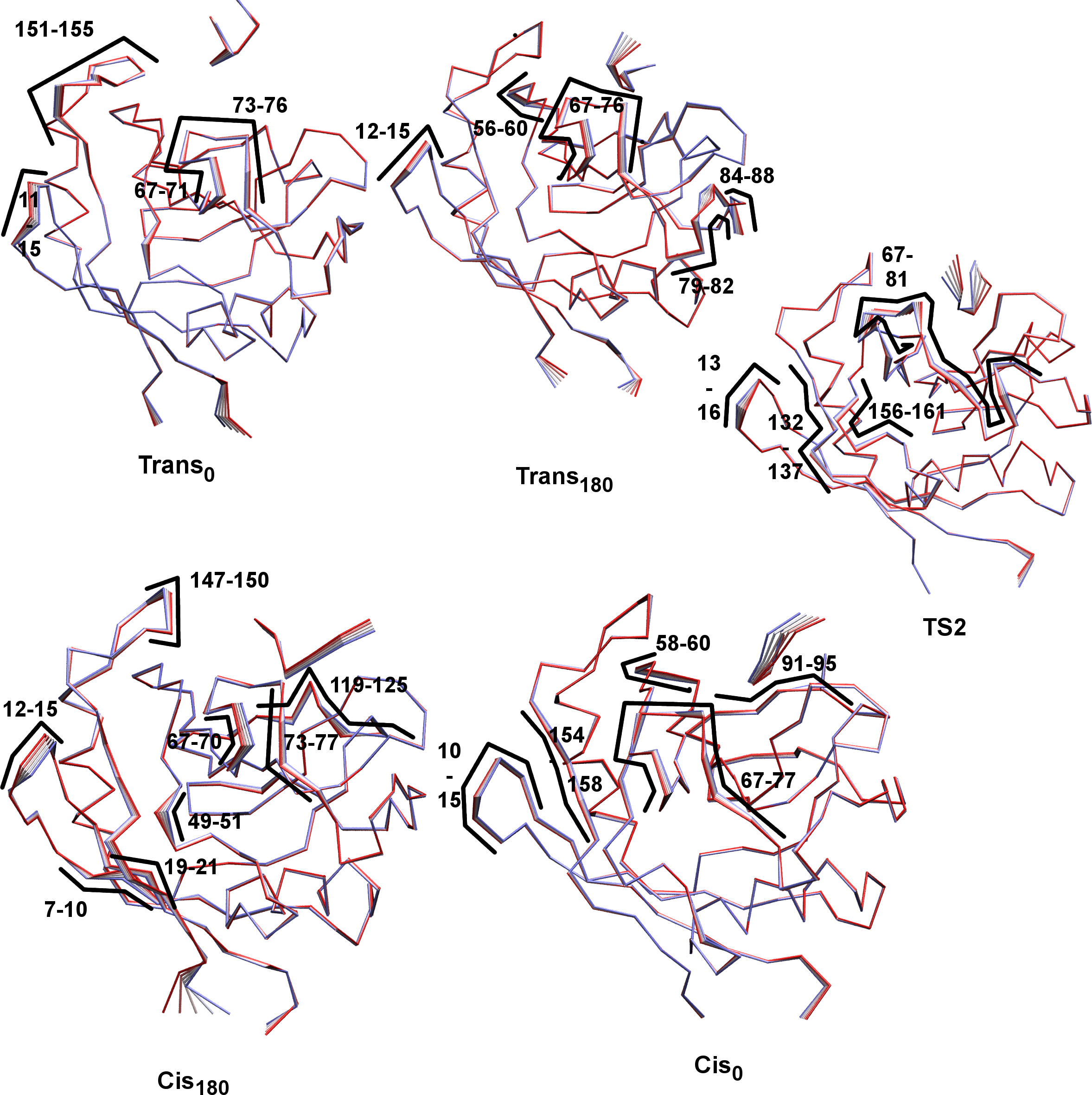

Supplement: Figure S6 — Normal modes for fluctuations observed on each minimum and the catalytic TS (TS2) of the enzyme catalyzed /HAGPIA/ isomerization. The modes are calculated from principal component analysis (PCA). Relevant modes (high eigenvalue) that are similar to fluctuations reported previously [15] are displayed. (2.01 MB TIF) [file pcbi.1000309.s014.tif]
