# Supplementary material for: Mechanism of Action of Cyclophilin A Explored by Metadynamics Simulations
Source: PLoS Comput Biol. 2009 Mar 13;5(3):e1000309. doi: 10.1371/journal.pcbi.1000309 (PMC2643488; doi:10.1371/journal.pcbi.1000309)
Supplement: Table S1 — Isomerase activity of several wild type cyclophilins and CypA mutants. (0.04 MB DOC) [file pcbi.1000309.s015.doc]

**Table S1.** Isomerase activity of several wild type cyclophilins and CypA mutants.

| Organism/Name used in publication | Exchange/Mutation | k_cat_/K_M_ (M^-1^s^-1^) | Substrate |
| --- | --- | --- | --- |
| *H. sapiens*/CypA | - | 1.6 10^7^ | Suc-AAPF-pNA |
| *C. elegans*/Cyp-9 | I57🡪V | 1.5 10^4^ | Suc-AAPF-pNA |
| *C.elegans*/Cyp-16 | I57🡪V | 2 10^3^ | Suc-ALPF-pNA |
| *R.norvegicus*/Matrin CYP | I57🡪V | 1.0 10^6^ | Suc-AAPF-pNA |
| *H.sapiens*/NKCR_HUMAN | I57🡪V | 7.5 10^5^ | Suc-AAPF-pNA |
| *S.cerevisiae*/Cpr7 | I57🡪V | 7 10^4^ ^a^ | Suc-AAPF-pNA |
| *D.melanogaster* | I57🡪V | 5.6 10^4^ | Suc-AAPF-pNA |
| *E.coli*/CypA | N102🡪T | 5.71 10^7^ | Suc-AAPF-pNA |
| *E.coli*/CypB | N102🡪T | 6.74 10^7^ | Suc-AAPF-pNA |
| *B.subtilis*/PpiB | N102🡪H | 1.1 10^6^ | Suc-AAPF-pNA |
| *L. pneumophilia*/Cyp18 | N102🡪R | 4.6 10^6^ | Suc-AAPF-pNA |
| *O.volvulus*/Cyp16 | N102🡪S | 5.2 10^2^ | Suc-ALPF-pNA |
| *H.sapiens*/CypA | H54Q | 2.4 10^6^ | Suc-AAPF-pNA |
| *H.sapiens*/CypA | R55A | 1.6 10^4^ | Suc-AAPF-pNA |
| *H.sapiens*/CypA | F60A | 5.1 10^4^ | Suc-AAPF-pNA |
| *H.sapiens*/CypA | Q111A^a^ | 2 4 10^6^ | Suc-AAPF-pNA |
| *H.sapiens*/CypA | F113A | 4.8 10^5^ | Suc-AAPF-pNA |
| *H.sapiens*/CypA | W121A | 1.4 10^6^ | Suc-AAPF-pNA |
| *H.sapiens*/CypA | H126Q | 8.4 10^4^ | Suc-AAPF-pNA |

1. Enzyme kinetic parameters
2. Circular dicroism of Q111A mutant showed that it is not propertly folded.

Adapted from [19,20]
