# Supplementary material for: Mechanism of Action of Cyclophilin A Explored by Metadynamics Simulations
Source: PLoS Comput Biol. 2009 Mar 13;5(3):e1000309. doi: 10.1371/journal.pcbi.1000309 (PMC2643488; doi:10.1371/journal.pcbi.1000309)
Supplement: Table S3 — Structural determinants and energetics of PEPT: averaged P4N….I5H-N H-bond number (NHB) and puckering (χ2 angle, Figure 2) within each region of the F(ζ, ψ) free energy profile. (0.03 MB DOC) [file pcbi.1000309.s017.doc]

|  | ψ | N_HB_ | χ2 angle | ∆F (kcal/mol) |
| --- | --- | --- | --- | --- |
| trans_0_ | ~0° | 0.99±0.04 | -1°±4° | 0 |
| trans_180_ | ~±180° | 0.010±0.006 | -2°±3° | 3 |
| Cis_0_ | ~0° | 0.98±0.04 | -25°±4° | 3 |
| Cis_180_ | ~±180° | 0.000±0.009 | -24°±3° | 5 |
| TS1 | ~0° | 0.99±0.004 | 7°±3° | 14 |
| TS2 | ~±180° | 0.3±0.1 | 6°±9° | 19 |
| TS3 | ~0° | 0.86±0.04 | 24°±2° | 18 |
| TS4 | ~±180° | 0.0+0.0 | -19°±4° | 20 |

**Table S3.** Structural determinants and energetics of PEPT: averaged P4N….I5H-N H-bond number (**N_HB_**) and puckering (χ2 angle, Chart 1) within each region of the F(ζ,ψ) free energy profile.
