# Supplementary material for: Mechanism of Action of Cyclophilin A Explored by Metadynamics Simulations
Source: PLoS Comput Biol. 2009 Mar 13;5(3):e1000309. doi: 10.1371/journal.pcbi.1000309 (PMC2643488; doi:10.1371/journal.pcbi.1000309)
Supplement: Table S4 — Potential energy barriers (kcal/mol) for N-Acetylproline methylamide isomerization at the B3LYP/6-31G(d) level of theory and with the Amber99 force field. (0.04 MB DOC) [file pcbi.1000309.s018.doc]

**Table S4.** Potential energy barriers (kcal/mol) for N-Acetylproline methylamide isomerization at the B3LYP/6-31G(d) level of theory and with the Amber99 force field.

| Method | B3LYP/6-31G(d)^a^ |  | Amber99 force field |  |
| --- | --- | --- | --- | --- |
| Pathway | $\Delta E_{trans\to cis}$ |  | $\Delta E_{trans\to cis}$^a^ |  |
| **trans_0_**↔TS1/TS3↔ **cis_0_** | -2.7 |  | -3.6 |  |
| trans_180_↔TS2/TS4↔ cis_180_ | 2.1 |  | 0.7 |  |
|  | $\Delta E^{\text{‡}}_{trans\to cis}$^b^ | $\Delta E^{\text{‡}}_{cis\to trans}$^c^ | $\Delta E^{\text{‡}}_{trans\to cis}$^b^ | $\Delta E^{\text{‡}}_{cis\to trans}$^c^ |
| trans_0_↔**TS1**↔ cis_0_ | 13.3 | 16.1 | 11.2 | 14.8 |
| trans_180_↔**TS2**↔ cis_180_ | 18.1 | 16.0 | 14.4 | 13.7 |
| trans_0_↔**TS3**↔ cis_0_ | 14.0 | 16.7 | 14.2 | 17.8 |
| trans_180_↔**TS4**↔ cis_180_ | 23.7 | 21.7 | 20.4 | 19.7 |

1. Potential energy differences between *trans* and *cis* minima.
2. Potential energy barrier in *trans*-to-*cis* direction
3. Potential energy barrier in *cis*-to-*trans* direction
