# Supplementary material for: Mechanism of Action of Cyclophilin A Explored by Metadynamics Simulations
Source: PLoS Comput Biol. 2009 Mar 13;5(3):e1000309. doi: 10.1371/journal.pcbi.1000309 (PMC2643488; doi:10.1371/journal.pcbi.1000309)
Supplement: Table S5 — ζ and ψ (Figure 2) values used in N-Acetylproline methylamide partial optimization at the B3LYP/6-31G(d) level of theory and with the Amber99 force field. Parameters of the NPro…H-Ni+1 hydrogen bond and potential energy of each structure are included. (0.05 MB DOC) [file pcbi.1000309.s019.doc]

| Method | B3LYP/6-31G(d) |  |  |  |  |  |
| --- | --- | --- | --- | --- | --- | --- |
|  | ζ | Ψ | N_Pro_….N_i+1_ (Å) | N_Pro_….H-N_i+1_ | NPA^a^ charge on N_Pro_ (a.u.) | ΔE^b^  (kcal/mol) |
| Trans_0_ | -169º | -36º | 2.9 | 107º | -0.51 | 2.7 |
| Trans_180_ | -152º | 110º | 3.4 | 87º | -0.49 | 1.3 |
| cis_0_ | 2º | 11º | 2.8 | 109º | -0.52 | 0.0 |
| cis_180_ | 5º | 142º | 3.6 | 72º | -0.49 | 3.4 |
| TS1 | -99º | -17º | 2.8 | 111º | -0.60 | 16.1 |
| TS2 | 93º | 134º | 3.6 | 74º | -0.58 | 19.4 |
| TS3 | 95º | -23º | 2.8 | 110º | -0.63 | 16.7 |
| TS4 | -96º | 150º | 3.7 | 72º | -0.56 | 25.0 |
| Method | Amber99 force field |  |  |  |  |  |
|  | ζ | Ψ | N_Pro_….N_i+1_ (Å) | N_Pro_….H-N_i+1_ | Force field charge on N_Pro_ (a.u.) | ΔE ^b^  (kcal/mol) |
| Trans_0_ | -169º | -36º | 2.9 | 104º | -0.42 | 3.6 |
| Trans_180_ | -152º | 110º | 3.4 | 79º | -0.42 | 4.7 |
| cis_0_ | 2º | 11º | 2.9 | 107º | -0.42 | 0.0 |
| cis_180_ | 5º | 142º | 3.7 | 73º | -0.42 | 5.4 |
| TS1 | -99º | -17º | 2.9 | 107º | -0.42 | 14.8 |
| TS2 | 93º | 134º | 3.6 | 73º | -0.42 | 19.1 |
| TS3 | 95º | -23º | 2.9 | 105º | -0.42 | 17.8 |
| TS4 | -96º | 150º | 3.7 | 72º | -0.42 | 25.1 |

**Table S5.** ζ and ψ (Chart 1) fixed values used in N-Acetylproline methylamide partial optimization at the B3LYP/6-31G(d) level of theory and with the Amber99 force field. Parameters of the N_Pro_…H-N_i+1_ hydrogen bond and potential energy of each structure are included.

1. Natural Population Analysis (Reed, A. E.; Curtiss, L. A.; Weinhold, F. (1998) Intermolecular Interactions from A Natural Bond Orbital, Donor-Acceptor Viewpoint. *Chem Rev.*, *88*: 899-926)
2. Potential energy differences of each minimum and TS respect to *cis_0_*.
