# Supplementary material for: Mechanism of Action of Cyclophilin A Explored by Metadynamics Simulations
Source: PLoS Comput Biol. 2009 Mar 13;5(3):e1000309. doi: 10.1371/journal.pcbi.1000309 (PMC2643488; doi:10.1371/journal.pcbi.1000309)
Supplement: Table S6 — Number of H-bonds between PEPT-CypA complex and water molecules (0.03 MB DOC) [file pcbi.1000309.s020.doc]

| **PEPT residue** | **trans_0_** | **cis_0_** | **trans_180_** | **cis_180_** | **TS1** | **TS2** | **TS3** | **TS4** |
| --- | --- | --- | --- | --- | --- | --- | --- | --- |
| **H1** | 0.96±0.02 | 1.08±0.01 | 1.49±0.02 | 1.43±0.04 | 1.17±0.02 | 1.39±0.02 | 1.23±0.01 | 1.43±0.01 |
| **A2** | 0.59±0.01 | 0.007±0.001 | 0.20±0.01 | 0.28±0.02 | 0.16±0.01 | 0.20±0.01 | 0.204±0.004 | 0.220±0.005 |
| **G3** | 0.13±0.09 | 0.0014±0.005 | 0.15±0.01 | 0.003±0.002 | 0.10±0.01 | 0±0 | 0.005±0.001 | 0.177±0.005 |
| **I5** | 0.025±0.004 | 0.044±0.003 | 0.29±0.01 | 0.001±0.001 | 0.006±0.001 | 0.33±0.01 | 0.013±0.001 | 0±0 |
| **A6** | 0±0 | 0.053±0.003 | 0.22±0.01 | 0.16±0.01 | 0.021±0.003 | 0.30±0.01 | 0.115±0.003 | 0±0 |

**Table S6.** Number of H-bonds between PEPT-CypA complex and water molecules
