# Supplementary material for: Mechanism of Action of Cyclophilin A Explored by Metadynamics Simulations
Source: PLoS Comput Biol. 2009 Mar 13;5(3):e1000309. doi: 10.1371/journal.pcbi.1000309 (PMC2643488; doi:10.1371/journal.pcbi.1000309)
Supplement: Table S7 — Average distances between G3@PEPT and L98S99@CypA (0.03 MB DOC) [file pcbi.1000309.s021.doc]

**Table S7.** Average distances between G3@PEPT and L98S99@CypA

| **CypA…PEPT distances** | **trans_0_** | **cis_0_** | **trans_180_** | **cis_180_** | **TS1** | **TS2** | **TS3** | **TS4** |
| --- | --- | --- | --- | --- | --- | --- | --- | --- |
| L98N…G3N | 21±1 | 18.4±0.4 | 16.5±0.4 | 15.4±0.3 | 18±1 | 14.9±0.3 | 15.3±0.5 | 16.8±0.3 |
| L98N…G3O | 20±1 | 18.1±0.4 | 15.9±0.4 | 13.4±0.3 | 17±1 | 12.7±0.3 | 12.8±0.4 | 15.2±0.3 |
| S99N…G3N | 19±1 | 15.3±0.4 | 13.9±0.4 | 12.3±0.3 | 16±1 | 11.8±0.3 | 12.3±0.5 | 13.9±0.3 |
| S99N…G3O | 18±1 | 15.2±0.4 | 13.5±0.4 | 10.2±0.3 | 15±1 | 9.7±0.3 | 9.8±0.4 | 12.6±0.3 |
