# Supplementary material for: Mechanism of Action of Cyclophilin A Explored by Metadynamics Simulations
Source: PLoS Comput Biol. 2009 Mar 13;5(3):e1000309. doi: 10.1371/journal.pcbi.1000309 (PMC2643488; doi:10.1371/journal.pcbi.1000309)
Supplement: Text S1 — Clustering, error evaluation, puckering of peptide in water and analysis of secondary free energy profiles obtained in the bias exchange metadynamics simulations. (0.10 MB DOC) [file pcbi.1000309.s022.doc]

**Mechanism of action of cyclophilin A explored by metadynamics simulations**

Vanessa Leone^1,2^, Gianluca Lattanzi^3,4^, Carla Molteni^5^, Paolo Carloni^1,2^.

^1^International School for Advanced Studies (SISSA), Trieste, Italy, ^2^IIT - Italian Institute of Technology and DEMOCRITOS, Trieste, Italy, ^3^University of Bari, Bari, Italy, ^4^TIRES and INFN, Bari, Italy, ^5^Department of Physics, King's College, London, United Kingdom.

**Supplementary information**

**Clustering.** Clusters of structures in the (ζ, ψ) space were produced [1] and then grouped in minima and TS regions with the following procedure (See S1):

1. For each structure with ζ’, ψ’ values, count the number of neighbors structures whose ζ,ψ values are (ζ’ - 2δs_ζ_) ≤ ζ ≤ (ζ’ + 2δs_ζ_), (ψ’ - 2δs_ψ_) ≤ ψ ≤ (ψ’ + 2δs_ψ_); where δs_ζ_ and δs_ψ_ are the width of the gaussian used in the metadynamics run (see Tab. S2) for the ζ, ψ collective variables (CVs), respectively.
2. Take the structure with the largest number of neighbors with all its neighbors, store it as the first cluster and eliminate them from the pool of structures. Repeat the procedure for the remaining structures until all of them are assigned to one cluster.
3. Estimate clusters population for each CV pairs.
4. Calculate the best estimate of the clusters population with a WHAM approach (from the last 12ns of the simulation) using the statistics of all CV pairs [2].
5. Define the following regions (R hereafter) and group clusters of structures: *cis_0_*: ζ [-50°,40°], ψ [-90°,50°]; *cis_180_*: ζ [-50°,40°], ψ [-180°,-150°][100°,180°]; *trans_0_*: ζ [-180°,-130°] [120°,180°], ψ [-90°,60°]; *trans_180_*: ζ [-180°,-150°][100°,180°], ψ [-180°,-150°] [100°,180°]; TS1: ζ [-100°,-70°], ψ [-60°,20°]; TS2: ζ [160°,100°], ψ [100°,150°]; TS3: ζ [70°,100°], ψ [-50°,10°]; TS4 (PEPT-WAT): ζ [-100°,-60°], ψ [170°,120°]; TS4 (PEPT-CypA) ζ [-100°,-60°], ψ [70°,120°].
6. Calculate R population by averaging the cluster population belonging to each R.

For PEPT-WAT, *trans_o_*, *trans_180_*, *cis_o_* and *cis_180_* were associated to 29, 5, 21 and 16 clusters respectively. For PEPT-CypA, these minima were identified by 16, 11, 6 and 18 clusters, respectively. Instead, TS regions were composed only of one cluster, as expected for states that occupy narrow regions (similar to saddle points in potential energy surface). RMSD within the minima and TSs is less than 2Å in both systems.

**Error evaluation.** Two main sources of error are present, one related to sampling limitations, the other to the inaccuracy of the force field [3]. These errors are evaluated here in the R regions of the minima and of the TS.

The statistical error associated with variable *a* (ensemble of points {a_0_}) was obtained by integrating out the *b,c,d*… variables from the free energy profiles *F*(*a,b),F(a,c),F(a,d),*…. for all *a* belonging to {a_0_}:

$F(a_{0})=-k_{B}T\ln\sum_{x} F(a_{0},x)$, (1)

where x=b,c,d… and k_B_ is Boltzmann’s constant and T is temperature. The largest difference between the calculated F(a_0_) for the value a_0_ defining the bottom of minimum well or the saddle point, provides their respective errors. The statistical error on the free energy differences between the minima and the absolute minima turned out to be of the order of 1 kcal/mol. The statistical error of the free energy for the TS turned out to be of the order of 1.5 kcal/mol.

Force field deficiencies may arise from several factors, including (i) its simplicity – which does not allow capturing accurately all the physical interactions in the systems, (ii) the fact that the potential has been tuned to reproduce experimental data for minimum energy structures – this may tend to increase errors in the transition state structures [3]. Here we have investigated the accuracy of the force-field used (Amber [4]) by QM methods. We compare the *cis*↔*trans* isomerization potential energy of a model system, a peptide model (N-acetyl proline methylamide) with the AMBER force field and with DFT at the B3LYP/6-31G(d) level of theory. We consider analogous minima and TSs to those in the F(ζ,ψ) free energy profile (Fig. 2) by fixing the dihedral angles ζ,ψ during optimization (Tab. S5).

The energy differences for *trans* and *cis* minima at ψ ~ 0° and ψ ~ ±180° (*trans_o_*-*cis_o_* and *trans_180_*-*cis_180_*) turned out to be similar (maximum difference ~1.5 kcal/mol, Tab. S4-5). For *trans_0_*→TS1/TS3→*cis_0_* pathways, the force field potential energy barriers turned out to be roughly similar to the DFT ones (within ~ 2 kcal/mol). Instead, *trans_180_*→TS2/TS4→*cis_180_* classical potential energy barriers are lower than quantum ones by ~ 4 kcal/mol (Tab. S4). This is due to a higher destabilization of *trans_180_* with respect to *cis_180_*, TS2 and TS4 (Tab. S4) in the Amber force field.

**Puckering of PEPT-WAT (conformations of the F(ζ,ψ) plot).** *Cis_o_* and *cis_180_*  turned out to be down puckered: the χ2 angle (Chart 1) features a two peaks distribution (bimodal) with a largest maximum at χ2 =-40° (Fig. S3, Tab. S3). This is explained by larger clashes in the up-puckered structures than in the down-puckered ones, as previously suggested. Instead, the *trans* conformations, which do not have such clashes, are a mixture of up and down puckered structures, with two equal maxima found for χ2 =-40° and χ2 =+40° (Fig. S3, Tab. S3). These results are fully consistent with the statistical distributions observed across Pro-containing proteins in the Brookhaven Protein Databank [5].

TS1, which is the lowest in energy, has an equal population of up and down puckering; TS3, the second, is up-puckered; TS2 displays an even up and down puckering, and TS4 is down-puckered (Fig. S3, Tab. S3). Consistently, our calculated free energy barriers for puckering turn out to be small (ΔF^‡planar^ _down→up_= ΔF^‡planar^ _up→down_= 2.1 kcal/mol, for calculation see methods). In addition, these low barriers (compared to 10-18 kcal/mol *cis/trans* isomerization barriers), suggest that puckering does not play an important role in isomerization.

Proline puckering may play a role in prolyl *cis/trans* isomerization, as suggested by quantum calculations of model peptide (i.e N-acetyl-L-Pro-N’-methylamide) [6]. Because of the approximations in our calculations, we cannot validate nor disprove the proposal.

**Free energy plots with different CVs**

*PEPT-WAT.*

The F(ζ,p) plot (Fig. S4A) illustrates the role of pyramidalization (see Materials and Methods) along *cis* ↔ *trans* isomerization; during this process, P4N may switch from an almost sp^2^ hybridization (resonance of the prolyl-peptidyl bond) in minima to sp^3^ (pyramidalization) in TS. *p* does not change significantly on passing from the minima (ζ ~ ±180° or ζ ~ 0°) to the TSs (ζ ~ ±90° ), suggesting that pyramidalization is not a crucial variable for the process. However, we cannot be conclusive on this point, as the force field might not describe accurately the pyramidalization, which is a typical quantum effect. The barriers of the favoured isomerization pathways turn out to be 16 and 12 kcal/mol for *trans*→*cis* and *cis*→*trans*, respectively.

The F(ζ, *s1*) profile (Fig. S4B) shows the role of the intramolecular H-bond formed by NP4. The CV *s1* is constructed so that *s1* ~1 when NP4 forms an H-bond. The lowest free energy barrier involves a TS at *s1* ~ 1 and ζ ~ -90° (Fig. S4B). This is consistent with the favored pathway along TS1, because this TS is located at ζ ~ -90° and NP4 forms a more persistent H-bond (P4N…I5N H-bond persistency is almost 1) than the other TSs. Isomerization barriers turn out to be 14 and 11 kcal/mol for *trans*→*cis* and *cis*→*trans*, respectively.

The F(ζ, *s2*) profile (Fig. S4C) is used to investigate the role of P4N-water H-bonding for the isomerization. This is described by the CV *s2*. *s2*~ 1 when P4N forms a H-bond with a water molecule. Minima and TS feature low values for this CV, therefore such role is excluded. The preferred barriers of the isomerization process turn out to be 15 and 11 kcal/mol for *trans*→*cis* and *cis*→*trans*, respectively.

The F(*s1*,*p*) and F(*s2*,*p*) profiles (Fig S4D, Fig S4E) illustrate the effect of pyramidalization on P4N H-bonding with PEPT (*s1*) and water (*s2*). As for F(ζ,p), we do not observe any effect of pyramidalization on the CVs *s1* and *s2*.

*PEPT-CypA*

Fig. S5A shows the F(ζ,*p*) profile for the enzyme catalyzed mechanism. As for PEPT-WAT, the pyramidalization does not show an important effect along PEPT-CypA isomerization. The lowest barriers are 15 kcal/mol and 11 kcal/mol for *trans*→*cis* and *cis*→*trans*, respectively.

The F(ζ,*s1*) profile (Fig. S5B) illustrates the role of intra- and intermolecular H-bonds formed by P4N, described by *s1*. The CV assumes values of 1 when P4N forms a H-bond. Intramolecular H-bonds with P4N are favored in PEPT-WAT isomerization, but pathways with no intramolecular H-bonds are also allowed. Instead, in PEPT-CypA we observe that *cis-trans* isomerization proceeds only at *s1*~1 (Fig. S5B), when P4N may form an H-bond with PEPT or CypA. However, inspection of the structures shows that there is not an H-bond formed on P4N (Tab. 4). Instead, other PEPT atoms, i.e. G3O form H-bonds with CypA (Tab. 4). This brings P4N closer to the enzyme, thus increasing *s1* to the value 1. The favored barriers for *trans*→*cis* and *cis*→*trans* are 14 and 11 kcal/mol, respectively.

The F(ζ,*s3*) and F(ζ,*s4*) profiles (Figs. S5C-D) show the role of hydrophobic interactions of G3P4@PEPT-CypA and H1A2I5A6@PEPT-CypA, respectively. These are described by the CVs *s3* and *s4.* These CVs take high values (ranging from 10 to 60) when PEPT-CypA forms several hydrophobic interactions. The high values of both *s3* and *s4* in the lowest-free energy isomerization pathway suggest that the process is promoted by persistent hydrophobic interactions in the enzyme-substrate complex. The lower pathways of the process are 13 and 11 kcal/mol for *trans*→*cis* and *cis*→*trans*, respectively.

The F(ζ,*s5*) profile (Fig S5E) illustrates the role of the interaction between the C-terminal of the /HAGPIA/ peptide and the residues L98 and S99, described by the CV *s5*. Previous NMR studies showed that L98 and S99 chemical shifts change during catalyzed *cis*🡪*trans* isomerization. Hence, it was proposed that C-terminal rotation of the substrate would bring residues of the substrate closer to L98@CypA and S99@CypA [7]. *s5* assumes high values when I5A6@PEPT and L98S99@CypA form hydrophobic interactions. The low value of *s5* in the F(ζ,*s5*) plot (Fig. S5E) suggests that this interaction is not crucial in the isomerization process. The *trans*→*cis* and *cis*→*trans* preferred barriers are 11 and 12 kcal/mol, respectively. Indeed, as shown by molecular dynamics [8], L98N@CypA...G3N/O@PEPT S99N@CypA...G3N/O@PEPT range from 10 Å – 20 Å (Tab. S7) and they are larger in *trans* than in *cis* and TSs conformations. Therefore, our results confirm that N-terminal rotation of prolyl bond from *cis*🡪*trans* is also in agreement with NMR data, as it was proposed by a previous theoretical work [8]: during this rotation the substrate moves away from L98 and S99 residues, changing their local chemical environment.

The F(ζ,*s6*) profile (Fig. S5F) takes into account the interaction of R55 with the peptide as well as with other CypA active site residues. This is described by the variable *s6.* This CV takes values ranging between 1 and 2 when R55 forms H-bonds. R55 is coordinated (*s6* ~ 2) at TSs with ζ ~ +90 (corresponding to a mixture of TS2 and TS3), and is not coordinated (*s6* ~ 0) at TSs with ζ ~ -90 (TS1 and TS4). R55 forms H-bonds to Q63@CypA and N149@CypA in TS2 and P4@PEPT in TS4 (Tab. 4). Thus, the active site conformation in the proximity of R55 discriminates the pathways along TSs at ζ ~ +90 from pathways along TSs at ζ ~ -90. The lowest barriers of isomerization are 13 and 8 kcal/mol for *trans*→*cis* and *cis*→*trans*, respectively.

**References**

1. Daura X, Gademann K, Jaun B, Seebach D, van Gunsteren WF, *et al*. (1999) Peptide folding: When simulation meets experiment. Angew Chem Int Ed Engl 38:236-240.

2. Marinelli F, Pietrucci F, Piana S, Laio A. (2008) A kinetic model of Trp-cage folding from multiple biased molecular dynamics simulations. Submitted.

3. McDowell SE, Špačková N, Šponer J, Walter NG (2007) Molecular Dynamics Simulations of RNA: An In Silico Single Molecule Approach. Biopolymers 85:169–184.

4. Wang JM, Cieplak P, Kollman PA (2000) How well does a restrained electrostatic potential (RESP) model perform in calculating conformational energies of organic and biological molecules? J Comput Chem 21:1049-1074.

5. Ho BK, Coutsias EA, Seok, C, Dill KA (2005) The flexibility in the proline ring couples to the protein backbone. Protein Sci 14:1011-1018.

6. Jhon JS, Kang YK (1999) Imide Cis-Trans Isomerization of *N*-Acetyl-*N*'-methyl proline amide and Solvent Effects. *J. Phys. Chem. A* 103:5436-5439

7. Eisenmesser EZ, Bosco DA, Akke M, Kern D (2002) Enzyme dynamics during catalysis. Science 295:1520-1523.

8. Trzesniak D, van Gunsteren WF (2006) Catalytic mechanism of cyclophilin as observed in molecular dynamics simulations: pathway prediction and reconciliation of X-ray crystallographic and NMR solution data. Protein Sci 15:2544-2551.
