# Supplementary material for: Mechanism of Action of Cyclophilin A Explored by Metadynamics Simulations
Source: PLoS Comput Biol. 2009 Mar 13;5(3):e1000309. doi: 10.1371/journal.pcbi.1000309 (PMC2643488; doi:10.1371/journal.pcbi.1000309)
Supplement: Supplementary file 17 [file pcbi.1000309.s016.doc]

Table S2. Collective variables (CVs) and parameters used in bias-exchange metadynamics calculations

CV	Definition	r_0 (Å)	n	m	δ
	**For both PEPT-WAT and PEPT-CypA**				
ζ	$C\alpha^{i-1}$-O^{i-1}-$C\delta^{i}$-$C\alpha^{i}$	-	-	-	0.15 rad.
ψ	N^{i}-$C\alpha^{i}$-C^{i}-N^{i+1}	-	-	-	0.2 rad.
p	distance of P4N from (C^{i-1}-$C\alpha^{i}$-$C\delta$) geometrical center				0.015 Å
	**Only PEPT-WAT**				
s1	$s1 = \sum_j \frac{1-\left(r_{P4N-j}/r_0\right)^n}{1-\left(r_{P4N-j}/r_0\right)^m}$<br>j = H-bond donors of residues H1, A2, G3, I5 and A6	3.2	8	13	0.06
s2	$s2 = \sum_j \frac{1-\left(r_{P4N}/r_0\right)^n}{1-\left(r_{P4N}/r_0\right)^m}$<br>j = O atoms of water molecules	3.2	6	18	0.04
	**Only PEPT-CypA**				
s1	$s1 = \sum_j \frac{1-\left(r_{P4N-j}/r_0\right)^n}{1-\left(r_{P4N-j}/r_0\right)^m}$<br>j = H-bond donors of /HAGPIA/ residues G3, I5 and CypA residues R55, Q63, H126 N102	3.2	8	13	0.03
s3	$s3 = \sum_{i,j} \frac{1-\left(r_{ij}/r_0\right)^n}{1-\left(r_{ij}/r_0\right)^m}$<br>i = non polar carbons of /HAGPIA/ residues G3 and P4<br>j = non polar carbons of CypA residues F60, M61, Q63, A101, N102, F113, L122, H126	4.0	8	13	1.4
s4	$s4 = \sum_{i,j} \frac{1-\left(r_{ij}/r_0\right)^n}{1-\left(r_{ij}/r_0\right)^m}$<br>i = non polar carbons of /HAGPIA/ residues H1, A2, I5, A6<br>j = non polar carbons of CypA residues F60 Q63, N71, G72, T73, N102, A103, W121, L122	4.0	8	13	2.0
s5	$s5 = \sum_{i,j} \frac{1-\left(r_{ij}/r_0\right)^n}{1-\left(r_{ij}/r_0\right)^m}$<br>i = non polar carbons of /HAGPIA/ residues I5, A6<br>j = non polar carbons of /HAGPIA/ residues L98, S99	4.0	8	13	0.003
s6	$s6 = \sum_j \frac{1-\left(r_{R55N\varepsilon-j}/r_0\right)^n}{1-\left(r_{R55N\varepsilon-j}/r_0\right)^m}$<br>j = H-bond donor/acceptor of /HAGPIA/CypA residues Q63, N149	3.2	8	13	0.15
